# SGLT2 inhibition eliminates senescent cells and alleviates pathological aging

**DOI:** 10.1038/s43587-024-00642-y

**Published:** 2024-05-30

**Authors:** Goro Katsuumi, Ippei Shimizu, Masayoshi Suda, Yohko Yoshida, Takaaki Furihata, Yusuke Joki, Chieh-Lun Hsiao, Liang Jiaqi, Shinya Fujiki, Manabu Abe, Masataka Sugimoto, Tomoyoshi Soga, Tohru Minamino

**Affiliations:** 1 Department of Cardiovascular Biology and Medicine, Juntendo University Graduate School of Medicine, Tokyo, Japan; 2 Department of Cardiovascular Medicine, Niigata University Graduate School of Medical and Dental Sciences, Niigata, Japan; 3 Department of Cardiovascular Aging, National Cerebral and Cardiovascular Center Research Institute, Osaka, Japan; 4 Department of Cardiovascular Medicine, National Cerebral and Cardiovascular Center, Osaka, Japan; 5 Department of Physiology and Biomedical Engineering, Mayo Clinic, Rochester, MN USA; 6 Department of Advanced Senotherapeutics, Juntendo University Graduate School of Medicine, Tokyo, Japan; 7 Department of Animal Model Development, Brain Research Institute, Niigata University, Niigata, Japan; 8 Molecular and Cellular Aging, Tokyo Metropolitan Institute for Geriatrics and Gerontology, Tokyo, Japan; 9 Institute for Advanced Biosciences, Keio University, Yamagata, Japan; 10 Human Biology-Microbiome-Quantum Research Center (WPI-Bio2Q), Keio University, Tokyo, Japan; 11 Japan Agency for Medical Research and Development-Core Research for Evolutionary Medical Science and Technology (AMED-CREST), Japan Agency for Medical Research and Development, Tokyo, Japan

**Keywords:** Diabetes, Experimental models of disease, Ageing

## Abstract

It has been reported that accumulation of senescent cells in various tissues contributes to pathological aging and that elimination of senescent cells (senolysis) improves age-associated pathologies. Here, we demonstrate that inhibition of sodium–glucose co-transporter 2 (SGLT2) enhances clearance of senescent cells, thereby ameliorating age-associated phenotypic changes. In a mouse model of dietary obesity, short-term treatment with the SGLT2 inhibitor canagliflozin reduced the senescence load in visceral adipose tissue and improved adipose tissue inflammation and metabolic dysfunction, but normalization of plasma glucose by insulin treatment had no effect on senescent cells. Canagliflozin extended the lifespan of mice with premature aging even when treatment was started in middle age. Metabolomic analyses revealed that short-term treatment with canagliflozin upregulated 5-aminoimidazole-4-carboxamide-1-β-d-ribofuranoside, enhancing immune-mediated clearance of senescent cells by downregulating expression of programmed cell death-ligand 1. These findings suggest that inhibition of SGLT2 has an indirect senolytic effect by enhancing endogenous immunosurveillance of senescent cells.

## Abstract (web summary)

Katsuumi, Shimizu, Suda et al. report that SGLT2 inhibition reduces the senescence burden and alleviates aging traits in mice. The authors demonstrate an indirect mechanism of senescent cell removal, through enhancing immunosurveillance.

## Main

Most somatic cells have a finite lifespan and following exhaustive rounds of replication or detection of certain damage can undergo a state of essentially irreversible growth arrest, termed cellular senescence. The accumulation of senescent cells is presumed to be involved in the development of age-associated diseases^1–3^. For example, the accumulation of senescent cells in human and murine atherosclerotic lesions has been suggested to cause vascular inflammation and dysfunction, and inhibition of senescence regulators p53 and p21 improves vascular dysfunction and suppresses the development of atherosclerosis^4–7^. Furthermore, it has been reported that accumulation of senescent cells in obese visceral adipose tissue increases adipose tissue inflammation and insulin resistance, and that deletion of p53 from adipose tissue protects against dietary metabolic dysfunction^8,9^. However, direct inhibition of senescence regulators can also promote tumorigenesis^10^, meaning that strategies to curb the deleterious effects of senescent cell accumulation should be carefully designed to maintain the protection against tumorigenesis. Recent studies have shown that in mice, elimination of senescent cells (senolysis) improves some of the pathological consequences of aging, such as cardiovascular disease, metabolic disease, renal dysfunction and bone loss, and extends the healthy lifespan without increasing the incidence of cancer^7,11–15^. Senolytic agents have been developed that suppress anti-apoptotic pathways in senescent cells, and these agents have been shown to improve age-associated pathology^14–16^. Caloric restriction is known to extend the lifespan of various organisms, and this longer survival is associated with decreased tissue accumulation of senescent cells^17^. Some studies have reported that SGLT2 inhibitors inhibit the accumulation of senescent cells^18,19^. Because inhibition of SGLT2 leads to a loss of calories by increasing the urinary excretion of glucose^20^, we speculated that SGLT2 inhibitors might have a senolytic effect on senescent cells. Therefore, the purpose of this study was to verify whether SGLT2 inhibitors induce senolysis and to elucidate the underlying mechanism of senolysis.

## Results

### Effects of SGLT2 inhibition on senescent cells

**Fig. 1:**
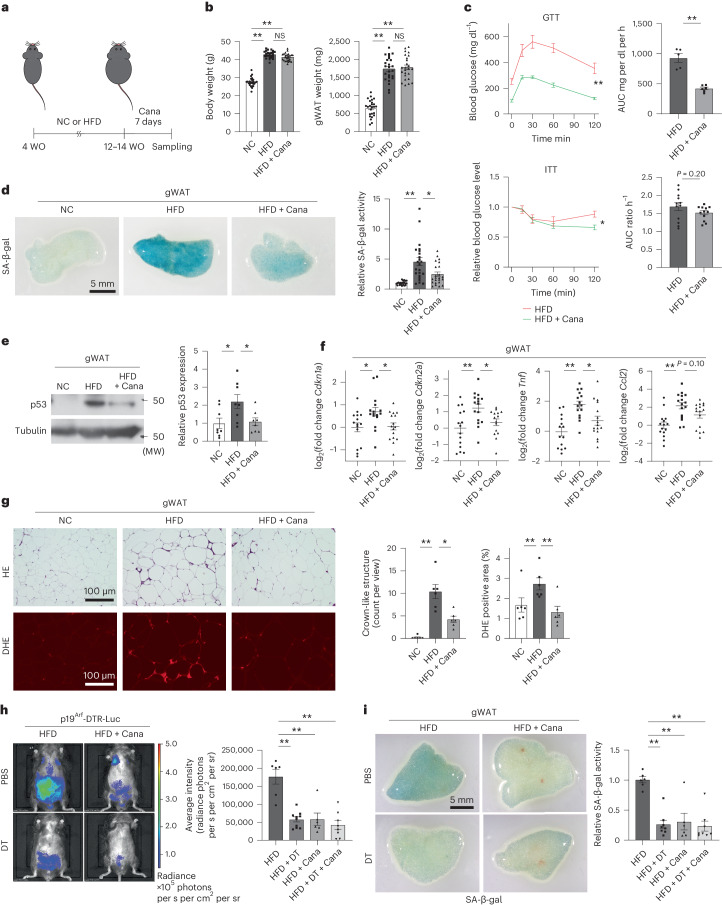
Effects of SGLT2 inhibition on senescent cells in visceral adipose tissue. **a**, Protocol of the experiments to test the senolytic effects of short-term treatment with canagliflozin (Cana). **b**, Body weight and gWAT weight of mice fed NC or HFD with or without canagliflozin (*n* = 25 each). **c**, GTT (*n* = 5, 6) and ITT (*n* = 12 each) of mice as prepared in **b**. GTT and ITT were performed from day 4 to day 7 after starting canagliflozin administration. **d**, SA-β-gal activity in gWAT of mice as prepared in **b** (*n* = 22, 23, 23). **e**, Western blot analysis for p53 in gWAT of mice as prepared in **b** (*n* = 8 each, from three gels/blots processed in parallel). **f**, qPCR analysis for *Cdkn1a*, *Cdkn2a*, *Tnf* and *Ccl2* in gWAT of mice as prepared in **b** (*n* = 15, 16, 16). **g**, Hematoxylin-eosin (HE) staining and dihydroethidium (DHE) assay in gWAT of mice as prepared in **b** (*n* = 6 each). **h**, p19^Arf^-dependent luciferase activity of mice on HFD after short-term administration of canagliflozin with or without DT treatment (*n* = 6, 10, 6, 8). **i**, SA-β-gal activity in gWAT of mice as prepared in **h** (*n* = 6, 8, 6, 8). Data were analyzed by two-way ANOVA followed by Tukey’s multiple comparison test (for equal variance) or Dunnett’s multiple comparison test (for unequal variance) (**b**,**d**–**i**), repeated measures analysis (**c**) or two-tailed unpaired Student’s *t*-test (**c**). *P < 0.05; **P < 0.01; NS, not significant. Exact *P* values: NC versus HFD <0.0001 (body weight and gWAT weight), NC versus HFD + Cana <0.0001 (body weight and gWAT weight), HFD versus HFD + Cana 0.0553 (body weight) and 0.8961 (gWAT weight) (**b**); HFD versus HFD + Cana 0.0003 (GTT-trend), 0.0015 (GTT-AUC), 0.0127 (ITT-trend) and 0.2013 (ITT-AUC) (**c**); NC versus HFD <0.0001, HFD versus HFD + Cana 0.0267 (**d**); NC versus HFD 0.0308, HFD versus HFD + Cana 0.0443 (**e**); NC versus HFD: 0.0272 (*Cdkn1a*), 0.0036 (*Cdkn2a*), <0.0001 (*Tnf*) and 0.0003 (*Ccl2*), HFD versus HFD + Cana 0.0353 (*Cdkn1a*), 0.0472 (*Cdkn2a*), 0.0219 (*Tnf*) and 0.1097 (*Ccl2*) (**f**); NC versus HFD 0.0038 (crown-like structure count) and 0.007 (DHE); HFD versus HFD + Cana 0.0255 (crown-like structure count) and 0.0042 (DHE) (**g**); HFD versus HFD + DT <0.0001, HFD versus HFD + Cana <0.0001, HFD versus HFD + DT + Cana <0.0001 (**h**); HFD versus HFD + DT <0.0001, HFD versus HFD + DT + Cana <0.0001 and HFD versus HFD + Cana 0.0002 (**i**). Data are shown as the mean ± s.e. in plots of all individual data (**b**–**i**) or as the mean ± s.e. in the spaghetti plot shown in Supplementary Fig. 1 (**c**). MW, molecular weight; WO, weeks old; AUC, area under the curve. Source data

**Extended Data Fig. 1:**
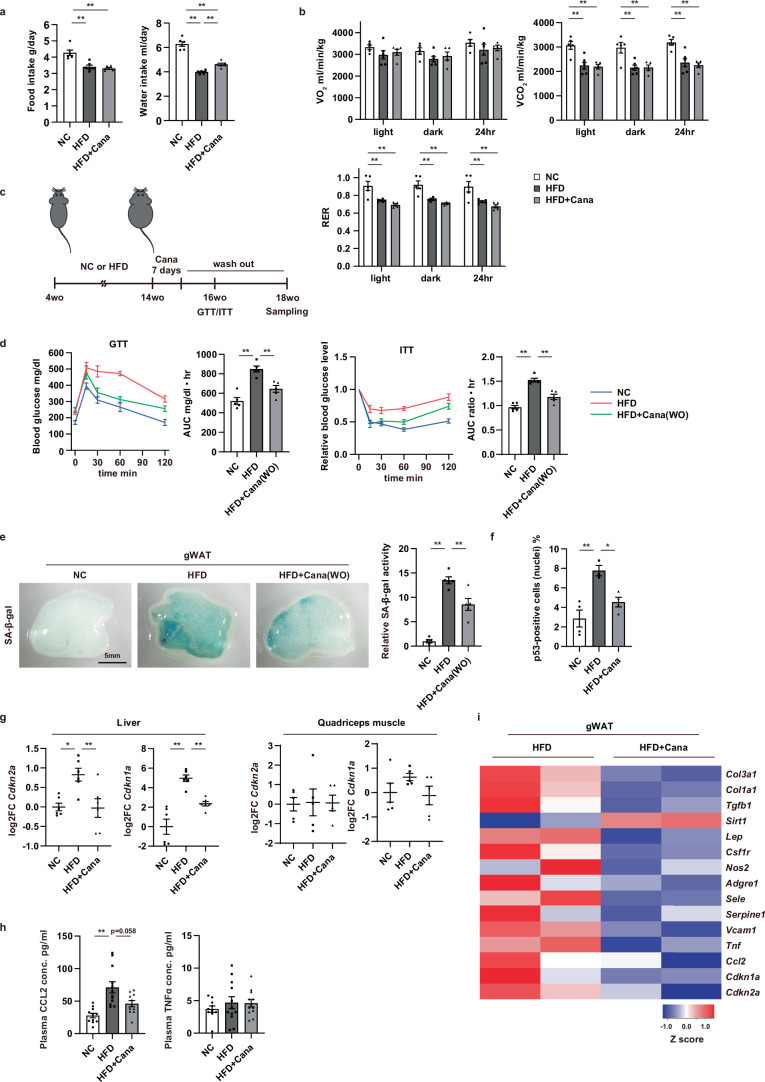
Effects of SGLT2 inhibition on senescent cells in visceral adipose tissue. **a**, Food intake of mice prepared according to the experimental protocol shown in Fig. 1a (n = 6 each). **b**, Oxygen consumption (VO2), CO2 emission (VCO2), and respiratory exchange ratio (RER) of mice as prepared in Extended Data Fig. 1a (n = 5, 6, 5). **c**, Protocol for performing the glucose tolerance test (GTT) and insulin tolerance test (ITT) after 1 week of canagliflozin administration followed by 1 week of no canagliflozin administration. NC, normal chow; HFD, high-fat diet. **d**, Glucose tolerance test (GTT) and insulin tolerance test (ITT) of mice as prepared in Extended Data Fig. 1c (n = 5 each). WO, wash out. **e**, SA-β-gal activity in gonadal white adipose tissue (gWAT) of mice as prepared in Extended Data Fig. 1c (n = 5 each). **f**, Immunostaining for p53 in gWAT as prepared in Extended Data Fig. 1a (n = 4, 3, 4). **g**, qPCR analysis for Cdkn1a and Cdkn2a in multiple organs of mice as prepared in Extended Data Fig. 1a (Liver, n = 6 each; Muscle, n = 5 each). **h**, ELISA for CCL2 (n = 11, 12, 11) and TNFα (n = 10, 12, 12) in the blood of mice as prepared in Extended Data Fig. 1a. **i**, RNA-sequence analysis for inflammatory molecules in gWAT of mice as prepared in Extended Data Fig. 1a (n = 2 each). Data were analyzed by two-way ANOVA followed by Tukey’s multiple comparison test (for equal variance) or Dunnett’s multiple comparison test (for unequal variance) (a, b, d–h). *P < 0.05, **P < 0.01. Exact P-value: NC versus HFD: 0.0003 (Food intake) and < 0.0001 (Water intake), NC versus HFD+Cana: < 0.0001 (Food intake and Water intake), HFD versus HFD+Cana: 0.008 (Water intake) (a); NC versus HFD: 0.0003 (VCO2-light and VCO2-24 hr), 0.0004 (VCO2-dark), 0.0006 (RER-light and RER-dark) and 0.0005 (RER-24 hr), NC versus HFD+Cana: 0.0003 (VCO2-light), 0.0007 (VCO2-dark), 0.0001 (VCO2-24 hr), < 0.0001 (RER-light, RER-dark and RER-24 hr) (b); NC versus HFD: < 0.0001 (GTT-trend, GTT-AUC, ITT-trend and ITT-AUC), HFD versus HFD+Cana(WO): 0.0086 (GTT-trend), 0.0029 (GTT-AUC), < 0.0001 (ITT-trend), 0.0004 (ITT-AUC) (d); NC versus HFD: < 0.0001, HFD versus HFD+Cana(WO): 0.0035 (e); NC versus HFD: 0.003, HFD versus HFD+Cana(WO): 0.0291 (f); NC versus HFD: 0.0122 (Liver-Cdkn2a) and 0.0017 (Liver-Cdkn1a), HFD versus HFD+Cana: 0.0097 (Liver-Cdkn2a) and 0.0003 (Liver-Cdkn1a) (g); NC versus HFD: 0.0009 (CCL2), HFD versus HFD+Cana: 0.0575 (CCL2) (h). Data are shown as the mean ± SE in plots of all individual data (a, b, d–h) or as the mean ± SE in the spaghetti plot shown in Supplementary Fig. 1 (d). Source data

**Extended Data Fig. 2:**
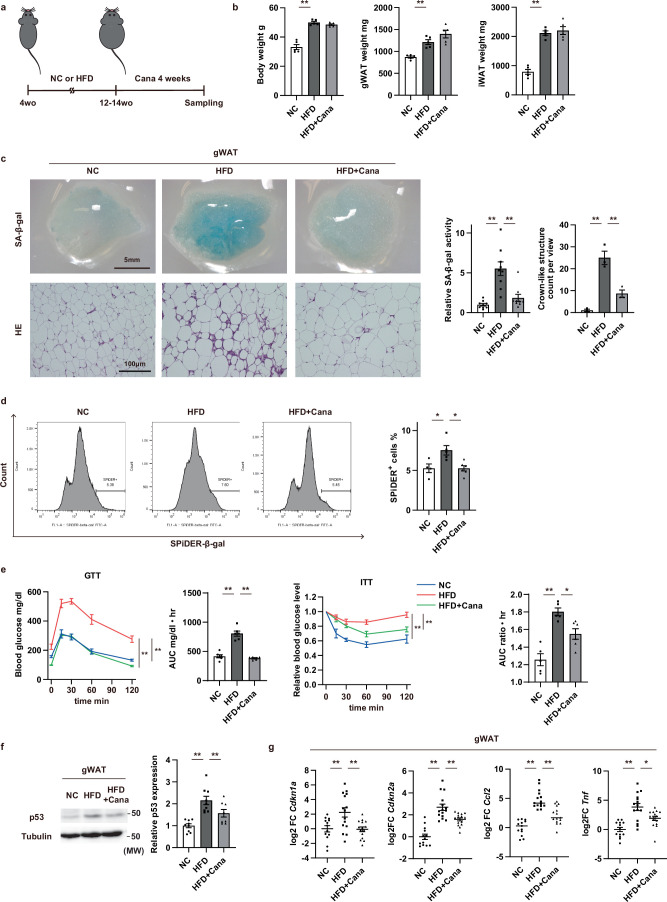
Effects of longer SGLT2 inhibition on senescent cells in visceral adipose tissue. **a**, Protocol of the experiments to test the senolytic effects of 4 weeks of treatment with canagliflozin (Cana). NC, normal chow; HFD, high-fat diet. **b**, Body weight and weight of gonadal white adipose tissue (gWAT) and inguinal WAT (iWAT) in mice fed NC or HFD with or without canagliflozin (n = 5 each). **c**, SA-β-gal activity (n = 9 each) and hematoxylin and eosin (HE) staining (n = 3 each) in gWAT of mice as prepared in Extended Data Fig. 2b. **d**, FACS analysis for SPiDERβ-gal + cells in gWAT of mice as prepared in Extended Data Fig. 2b (n = 4, 5, 6). **e**, Glucose tolerance test (n = 6 each) and insulin tolerance test (n = 5, 5, 6) of mice as prepared in Extended Data Fig. 2b. **f**, Western blot analysis for p53 in gWAT of mice as prepared in Extended Data Fig. 2b (n = 9, 9, 8 from 3 gels/blots processed in parallel). **g**, qPCR analysis for Cdkn1a, Cdkn2a, Tnf, and Ccl2 in gWAT of mice as prepared in Extended Data Fig. 2b (n = 13,15,15). Data were analyzed by two-way ANOVA followed by Tukey’s multiple comparison test (for equal variance) or Dunnett’s multiple comparison test (for unequal variance) (b–g) or repeated measures analysis (e). *P < 0.05, **P < 0.01. Exact P-value: NC versus HFD: 0.0003 (Body weight), 0.0053 (gWAT weight) and < 0.0001 (iWAT weight), NC versus HFD+Cana: 0.001 (Body weight), 0.0002 (gWAT weight) and < 0.0001 (iWAT weight) (b); NC versus HFD: 0.0016 (SA-β-gal activity) and 0.0004 (Crown-like structure count), HFD versus HFD+Cana: 0.0071 (SA-β-gal activity) and 0.0031 (Crown-like structure count) (c); NC versus HFD: 0.0219, HFD versus HFD+Cana: 0.0119 (d); NC versus HFD: < 0.0001 (GTT-trend, ITT-trend, and ITT-AUC) and 0.0002 (GTT-AUC), HFD versus HFD+Cana: < 0.0001 (GTT-trend), 0.0002 (GTT-AUC), 0.0013 (ITT-trend) and 0.0228 (ITT-AUC) (e); NC versus HFD: < 0.0001, HFD versus HFD+Cana: 0.039 (f); NC versus HFD: 0.0228(Cdkn1a) and < 0.0001 (Cdkn2a, Ccl2, and Tnf), HFD versus HFD+Cana: 0.0122 (Cdkn1a), 0.0075 (Cdkn2a), < 0.0001 (Ccl2) and 0.013 (Tnf) (g). Data are shown as the mean ± SE in plots of all individual data (b–g) or as the mean ± SE in the spaghetti plot shown in Supplementary Fig. 1 (e). Gating strategy in FACS analysis was shown in Supplementary Fig. 3c (d). Source data

To test whether SGLT2 inhibitors affect the senescent cell burden in vivo, mice were fed a high-fat diet (HFD) for 8–10 weeks and treated with the SGLT2 inhibitor canagliflozin for 7 days (Fig. 1a). Short-term treatment with canagliflozin did not affect body weight, weight of gonadal white adipose tissue (gWAT), food intake or oxygen consumption, but significantly improved insulin resistance and glucose intolerance compared with the control HFD group (Fig. 1b,c, Extended Data Fig. 1a,b and Supplementary Fig. 1a). We also evaluated glucose metabolism after 1 week of canagliflozin administration followed by 1 week of no canagliflozin administration. Even when canagliflozin was considered to be sufficiently washed out, a significant improvement in glucose metabolism was maintained compared with the control HFD group (Extended Data Fig. 1c,d and Supplementary Fig. 1b). Canagliflozin treatment also led to a significant improvement in HFD-induced senescence-like changes in gWAT and other tissues (for example, liver), including reduction of senescence-associated β-galactosidase (SA-β-gal) activity and decreased expression of negative cell-cycle regulators compared with the control HFD group (Fig. 1d–f and Extended Data Fig. 1e–g). Treatment with canagliflozin resulted in a statistically significant reduction or trend toward a reduction in the expression of proinflammatory senescence-associated secretory phenotype (SASP) factors (Fig. 1f and Extended Data Fig. 1h,i). We also found that canagliflozin treatment caused a decrease in crown-like structures (indicators of adipose tissue inflammation) and reduced oxidative stress (Fig. 1g). Furthermore, 4 weeks of treatment with canagliflozin showed more-pronounced improvement of senescence-like phenotypes, such as decreased SA-β-gal activity and decreased expression of negative cell-cycle regulators in gWAT, without reducing body weight or gWAT weight, and achieved significant improvement of adipose tissue inflammation, attenuating metabolic abnormalities induced by HFD (Extended Data Fig. 2a–g and Supplementary Fig. 1c).

To further investigate the potential senolytic effect of canagliflozin, we used a transgenic mouse model expressing the diphtheria toxin (DT) receptor (human HB-EGF I117V/L148V) and luciferase at the *Cdkn2A* locus (p19 alternative reading frame (p19^Arf^)–diphtheria toxin receptor (p19^Arf^–DTR) mouse)^21^. In this model, p19^Arf^-expressing cells could be eliminated by administration of DT, and the presence of these cells could be assessed by monitoring luciferase activity. p19^Arf^–DTR mice were fed HFD for 8 weeks and then administered canagliflozin for 7 days with or without DT treatment. In vivo fluorescence imaging analysis revealed that HFD-induced accumulation of p19^Arf^-expressing cells was significantly attenuated to the same extent by both canagliflozin and DT (Fig. 1h). DT had no significant additive effect on canagliflozin (Fig. 1h), supporting the effectiveness of canagliflozin in eliminating senescent cells. Consistent with the results of the in vivo fluorescence imaging analyses, canagliflozin attenuated SA-β-gal activity in gWAT, and no significant additive effects of DT were observed in mice treated with canagliflozin (Fig. 1i).

### Effects of insulin treatment on senescent cells

**Extended Data Fig. 3:**
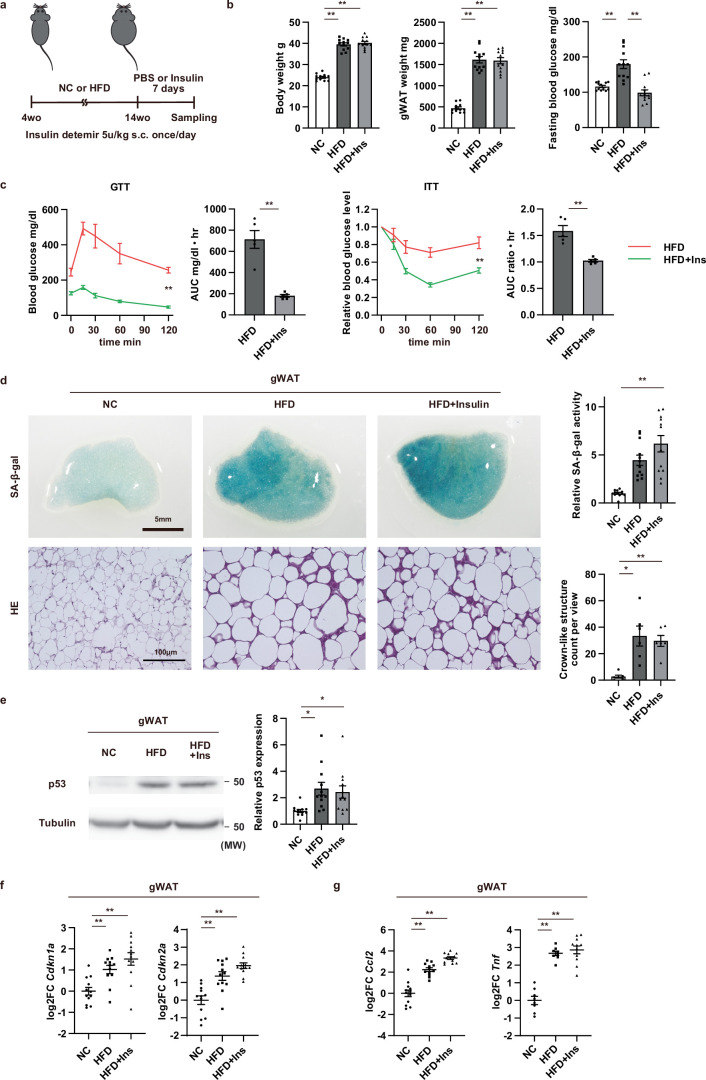
Effects of insulin treatment on senescent cells in visceral adipose tissue. **a**, Protocol of the experiments to test the senolytic effects of short-term treatment with insulin. NC, normal chow; HFD, high-fat diet. **b**, Body weight and gonadal white adipose tissue (gWAT) weight of mice fed NC or HFD with or without insulin (Ins) (n = 12 each). **c**, Glucose tolerance test and insulin tolerance test of mice as prepared in Extended Data Fig. 3b (n = 6 each). **d**, SA-β-gal activity (n = 12 each) and hematoxylin and eosin (HE) staining (n = 6 each) in gWAT of mice as prepared in Extended Data Fig. 3b. **e**, Western blot analysis for p53 in gWAT of mice as prepared in Extended Data Fig. 3b (n = 12 each from 4 gels/blots processed in parallel). **f**, qPCR analysis for Cdkn1a and Cdkn2a in gWAT of mice as prepared in Extended Data Fig. 3b (n = 12 each). **g**, qPCR analysis for Ccl2 and Tnf in gWAT of mice as prepared in Extended Data Fig. 3b (n = 12 each). Data were analyzed by two-way ANOVA followed by Tukey’s multiple comparison test (for equal variance) or Dunnett’s multiple comparison test (for unequal variance) (b, d–g), repeated measures analysis (c), or two-tailed unpaired Student’s *t*-test (c). *P < 0.05, **P < 0.01. Exact P-value: NC versus HFD: < 0.0001 (Body weight and gWAT weight) and 0.0011 (Fasting blood glucose), NC versus HFD+Ins: < 0.0001 (Body weight and gWAT weight), HFD versus HFD+Ins: 0.0001 (Fasting blood glucose) (b); HFD versus HFD+Ins: < 0.0001 (GTT-trend and ITT-trend), 0.0029 (GTT-AUC) and 0.0044 (ITT-AUC) (c); NC versus HFD: 0.0001 (SA-β-gal activity) and 0.0262 (Crown-like structure count), NC versus HFD+Ins: 0.0003 (SA-β-gal activity) and 0.0022 (Crown-like structure count) (d); NC versus HFD: 0.0152, NC versus HFD+Ins: 0.033 (e); NC versus HFD: 0.0092 (Cdkn1a) and 0.0003 (Cdkn2a), NC versus HFD+Ins: 0.0001 (Cdkn1a) and < 0.0001 (Cdkn2a) (f); NC versus HFD: < 0.0001 (Ccl2 and Tnf), and NC versus HFD+Ins: < 0.0001 (Ccl2 and Tnf) (g). Data are shown as the mean ± SE in plots of all individual data (b–g) or as the mean ± SE in the spaghetti plot shown in Supplementary Fig. 1 (c). Source data

**Extended Data Fig. 4:**
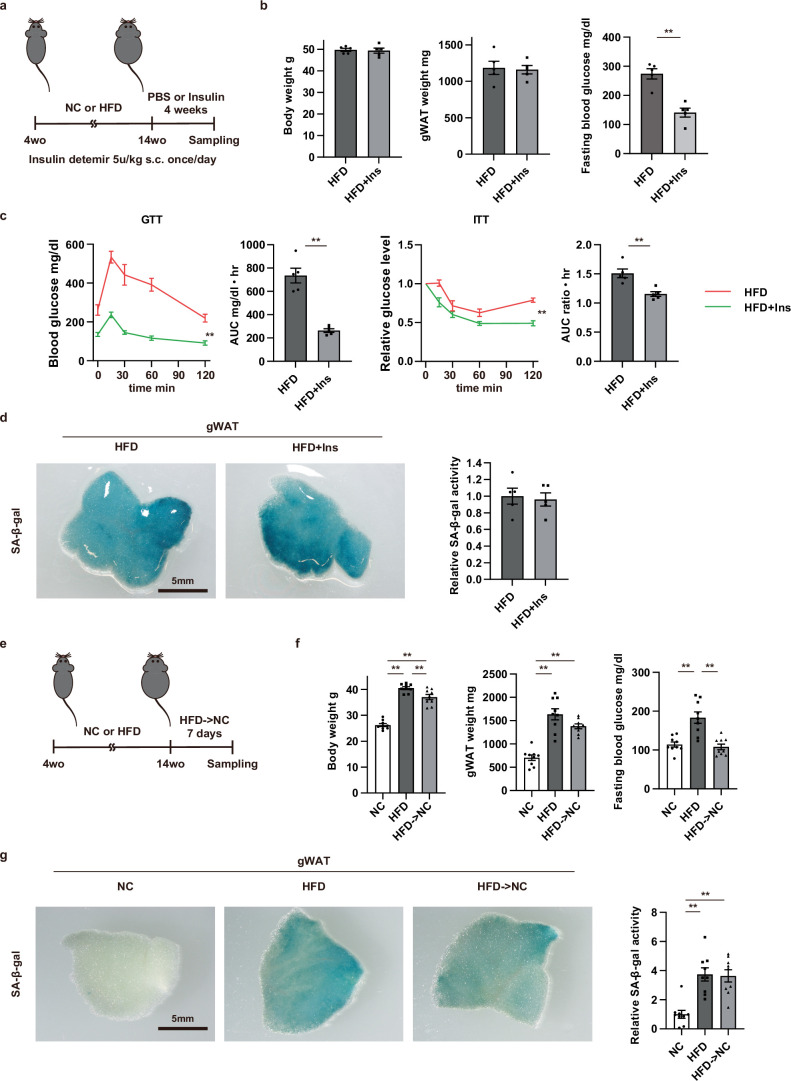
Effects of normalization of glucose metabolism on senescent cells in visceral adipose tissue. **a**, Protocol of the experiments to test the senolytic effects of 4 weeks of treatment with insulin. NC, normal chow; HFD, high-fat diet. **b**, Body weight, gonadal white adipose tissue (gWAT) weight, and fasting blood glucose of mice fed NC or HFD with or without insulin (Ins) (n = 5 each). **c**, Glucose tolerance test and insulin tolerance test in mice as prepared in Extended Data Fig. 4b (n = 5 each). **d**, SA-β-gal activity in gWAT of mice as prepared in Extended Data Fig. 4b (n = 5 each). **e**, Protocol of the experiments to test the senolytic effects of returning to NC after receiving HFD. NC, normal chow; HFD, high-fat diet. **f**, Body weight, gWAT weight, and fasting blood glucose of mice prepared according to the protocol shown in Extended Data Fig. 4e (n = 9 each). **g**, SA-β-gal activity in gWAT of mice as prepared in Extended Data Fig. 4f (n = 9 each). Data were analyzed by two-tailed unpaired Student’s *t*-test (b–d) or two-way ANOVA followed by Tukey’s multiple comparison test (for equal variance) or Dunnett’s multiple comparison test (for unequal variance) (f, g) or repeated measures analysis (c). *P < 0.05, **P < 0.01. Exact P-value: HFD versus HFD+Ins: 0.0005 (Fasting blood glucose) (b); HFD versus HFD+Ins: < 0.0001 (GTT-trend), 0.0029 (GTT-AUC), 0.0009 (ITT-trend) and 0.0058 (ITT-AUC) (c); NC versus HFD: < 0.0001 (Body weight and gWAT weight) and 0.0002 (Fasting blood glucose), NC versus HFD- > NC: <0.0001 (Body weight and gWAT weight), HFD versus HFD- > NC: 0.0073 (Body weight) and < 0.0001 (Fasting blood glucose) (f); NC versus HFD: 0.0001, and NC versus HFD- > NC: 0.0002 (g). Data are shown as the mean ± SE in plots of all individual data (b–d, f, g) or as the mean ± SE in the spaghetti plot shown in Supplementary Fig. 1 (c). Source data

Short-term treatment with canagliflozin may eliminate senescent cells by lowering blood glucose^22^. To test this possibility, we fed mice HFD for 8–10 weeks, administered insulin for 1 week, which induced metabolic improvements comparable with those induced by canagliflozin treatment, and observed the effects on senescent cells (Extended Data Fig. 3a). The results showed that short-term insulin treatment improved glucose intolerance and insulin resistance without affecting body weight or gWAT weight in these mice compared with the control HFD group (Extended Data Fig. 3b,c and Supplementary Fig. 1d). In contrast to short-term treatment with canagliflozin, short-term treatment with insulin did not improve HFD-induced senescence-like changes or inflammation in gWAT (Extended Data Fig. 3d–g). Likewise, 4 weeks of insulin treatment did not affect HFD-induced upregulation of SA-β-gal activity in gWAT, body weight or gWAT weight, although glucose intolerance and insulin resistance were significantly improved compared with the control HFD group (Extended Data Fig. 4a–d and Supplementary Fig. 1e).

We also examined whether normalizing the metabolic system by returning HFD-fed mice to a normal chow (NC) would affect the accumulation of senescent cells in gWAT (Extended Data Fig. 4e). Returning the HFD-fed mice to NC for 7 days improved plasma glucose levels and slightly decreased body weight but did not affect gWAT weight (Extended Data Fig. 4f). More importantly, returning the HFD-fed mice to NC did not improve HFD-induced upregulation of SA-β-gal activity in gWAT compared with the control HFD group (Extended Data Fig. 4g), suggesting that the effects of canagliflozin were not attributable to normalization of glucose metabolism.

### Mechanism of the senolytic effects of SGLT2 inhibition

**Fig. 2:**
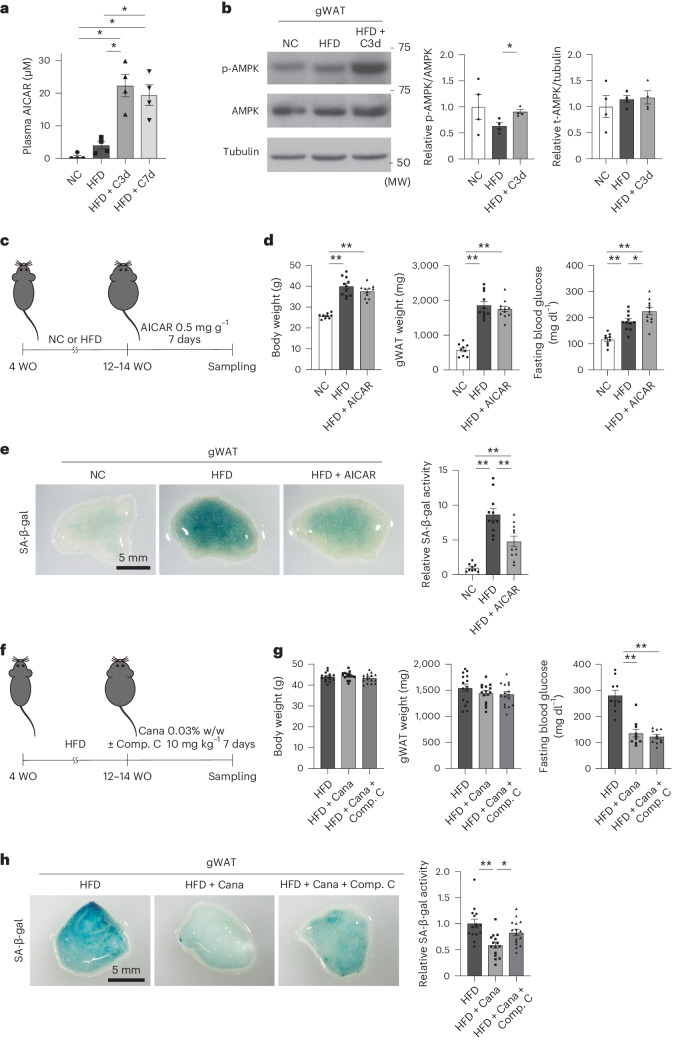
Potential mechanism of the senolytic effects of SGLT2 inhibition. **a**, Plasma AICAR level of mice fed NC or HFD with or without canagliflozin (*n* = 4 each). C3d, canagliflozin treatment for 3 days; C7d, canagliflozin treatment for 7 days. **b**, Western blot analysis for p-AMPK, AMPK and tubulin in gWAT of mice as prepared in **a** (*n* = 4 each from 2 gels/blots processed in parallel). **c**, Protocol of the experiments to test the senolytic effects of short-term treatment with AICAR. **d**, Body weight, gWAT weight and fasting blood glucose of mice fed NC or HFD with or without AICAR (*n* = 9, 11, 11). **e**, SA-β-gal activity in gWAT of mice as prepared in **d** (*n* = 9, 11, 11). **f**, Protocol of the experiments to test the effects of Compound C (Comp. C) on canagliflozin treatment. **g**, Body weight, gWAT weight and fasting blood glucose of HFD-fed mice after canagliflozin treatment with or without Comp. C (*n* = 15, 15, 16). **h**, SA-β-gal activity in gWAT of mice as prepared in **g** (*n* = 15, 15, 16). The data were analyzed by two-way ANOVA followed by Tukey’s multiple comparison test (for equal variance) or Dunnett’s multiple comparison test (for unequal variance) (**a**,**b**,**d**,**e**,**g**,**h**). *P < 0.05, **P < 0.01. Exact *P* values: NC versus HFD + C3d 0.0317, NC versus HFD + C7d 0.0371, HFD versus HFD + C3d 0.0322, HFD versus HFD + C7d 0.0443 (**a**); HFD versus HFD + C3d 0.038 (p-AMPK/AMPK) (**b**); NC versus HFD <0.0001 (body weight and gWAT weight) and 0.0007 (fasting blood glucose), NC versus HFD + AICAR <0.0001 (body weight, gWAT weight and fasting blood glucose), HFD versus HFD + Cana 0.0457 (fasting blood glucose) (**d**); NC versus HFD <0.0001, NC versus HFD + AICAR 0.0014, HFD versus HFD + Cana 0.0076 (**e**); HFD versus HFD + Cana <0.0001 (fasting blood glucose), HFD versus HFD + Cana + Comp. C <0.0001 (fasting blood glucose) (**g**); HFD versus HFD + Cana 0.0002, and HFD + Cana versus HFD + Cana + Comp. C 0.0346 (**h**). Data are shown as the mean ± s.e. in plots of all individual data (**a**,**b**,**d**,**e**,**g**,**h**). t-AMPK, total AMPK. Source data

**Extended Data Fig. 5:**
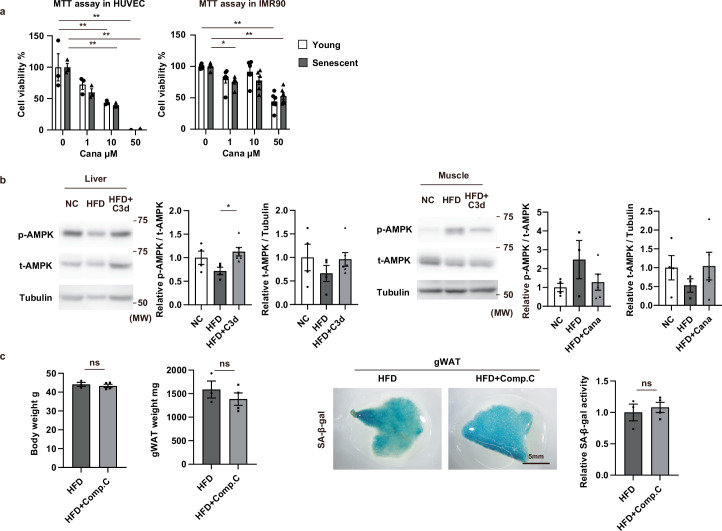
Potential mechanism of the senolytic effects of SGLT2 inhibition. **a**, MTT assay in human umbilical vein endothelial cells (HUVEC, young vs. replicative senescent cells, n = 3 each) or human fibroblasts (IMR90, young vs. irradiation-induced senescent cells, n = 6 each) treated with canagliflozin (Cana) for 48 hours. **b**, Western blot analysis for phospho-AMP-activated protein kinase (p-AMPK), AMPK, and tubulin in multiple organs of mice as prepared in Fig. 2a (Liver, n = 4, 4, 6 from 2 gels/blots processed in parallel; Muscle, n = 4, 3, 5 from 2 gels/blots processed in parallel). **c**, Body weight, gonadal white adipose tissue (gWAT) weight, and SA-β-gal activity in gWAT of HFD-fed mice after treatment with or without Comp C (n = 3, 4). Data were analyzed by two-way ANOVA followed by Tukey’s multiple comparison test (a, b) or two-tailed unpaired Student’s *t*-test (c). *P < 0.05; **P < 0.01; ns, not significant. Exact P-value: Young HUVEC: 0.0058 (0 μM versus 10 μM) and < 0.0001 (0 μM versus 50 μM), Senescent HUVEC: 0.0032 (0 μM versus 10 μM) and < 0.0001 (0 μM versus 50 μM), Young IMR90: < 0.0001 (0 μM versus 50 μM), and Senescent IMR90: 0.035 (0 μM versus 1 μM) and < 0.0001 (0 μM versus 50 μM) (a); HFD versus HFD+C3d: 0.0408 (Liver-pAMPK/tAMPK) (b); HFD versus HFD+Comp.C: 0.5899 (Body weight) and 0.3911 (gWAT weight) (c). Data are shown as the mean ± SE in plots of all individual data (a–c). Source data

To test whether a SGLT2 inhibitor might have a direct impact on senescent cells, we treated young human cells and senescent cells with canagliflozin; even at high drug concentrations (Supplementary Fig. 2a,b and Supplementary note 1), we found no direct senolytic effect on senescent cells (Extended Data Fig. 5a). To investigate the potential mechanisms of SGLT2 inhibition-induced senolysis, we next performed a metabolomic analysis in the HFD-induced obesity model after treatment with canagliflozin. We found that SGLT2 inhibition significantly increased the plasma level of 5-aminoimidazole-4-carboxamide-1-β-d-ribofuranoside (AICAR), a metabolite well known to activate AMP-activated protein kinase (AMPK) (Fig. 2a and Supplementary Table 1). Consistent with this, phospho-AMPK (p-AMPK) levels were upregulated in gWAT and liver by SGLT2 inhibition compared with the control HFD group (Fig. 2b and Extended Data Fig. 5b).

To test whether AICAR is involved in senolysis induction by canagliflozin, mice were fed HFD for 8–10 weeks and then treated with AICAR for 1 week (Fig. 2c). Treatment with AICAR did not change body weight or gWAT weight, but it did increase fasting blood glucose compared with the control HFD group (Fig. 2d). AICAR treatment significantly reduced SA-β-gal activity in gWAT compared with the control HFD group (Fig. 2e). Next, we tested whether suppression of AMPK activity inhibited the reduction in senescence load induced by SGLT2 inhibition. Mice were fed HFD for 8–10 weeks and then treated with canagliflozin and Compound C, an AMPK inhibitor, for 1 week (Fig. 2f). The results showed that additional treatment with Compound C did not change body weight, gWAT weight or fasting blood glucose, but did significantly increase SA-β-gal activity in gWAT compared with the canagliflozin-treated HFD group (Fig. 2g,h and Extended Data Fig. 5c). These results suggest that canagliflozin increases AICAR levels and that this effect may be responsible for the indirect senolytic effect of canagliflozin.

### SGLT2 inhibition affects programmed cell death-ligand 1 expression by senescent cells

Senescent cells that accumulate in tissues activate the immune system by secreting SASP factors. The activated immune system is believed to maintain tissue homeostasis by removing senescent cells^3^. In aging and pathological conditions, this removal mechanism becomes dysfunctional (anergy) and senescent cells continue to accumulate in tissues; the increased secretion of SASP factors triggers chronic inflammation, which is thought to be involved in the development of aging-related diseases^23,24^. As an immune checkpoint molecule, programmed cell death-1 (PD-1)/programmed cell death-ligand 1 (PD-L1) is known to negatively regulate activation of the immune system against tumors^25^. A recent study reported that PD-L1 is expressed in some populations of senescent cells and suppresses the senescent cell removal system^26^. The study also showed that senescent cells with higher expression of PD-L1 have higher expression of SASP factors and are more strongly involved in pathological aging phenotypes^26^.

**Fig. 3:**
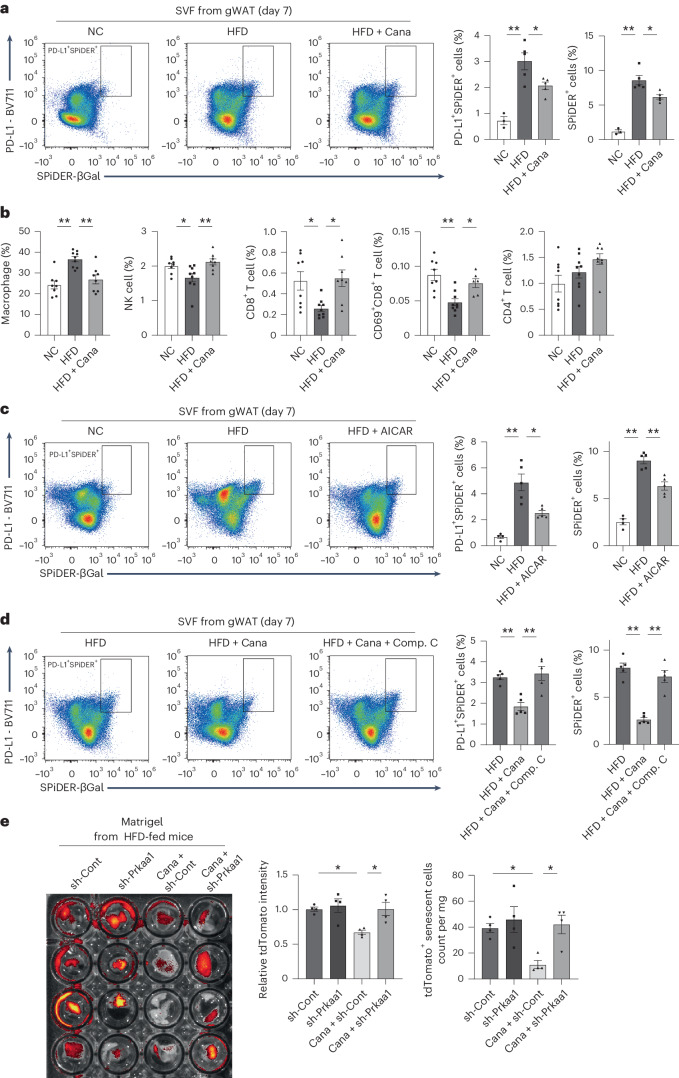
Effects of SGLT2 inhibition on PD-L1 expression by senescent cells. **a**, FACS analysis for PD-L1^+^SPiDER-βGal^+^ cells in stromal vascular fraction (SVF) obtained from gWAT of mice fed NC or HFD with or without canagliflozin (Cana) (*n* = 3, 5, 5) on day 7. **b**, FACS analysis for immune cells in gWAT of mice as prepared in **a** (*n* = 8, 9, 8) on day 7. **c**, FACS analysis for PD-L1^+^SPiDER-βGal^+^ cells in SVF from gWAT of mice fed NC or HFD with or without AICAR (*n* = 4, 5, 5) on day 7. **d**, FACS analysis for PD-L1^+^SPiDER-βGal^+^ cells in SVF from gWAT of HFD-fed mice after canagliflozin treatment with or without Compound C (Comp. C) (*n* = 5 each) on day 7. **e**, Matrigel transplantation model containing senescent cells (*n* = 4 each). Data were analyzed by two-way ANOVA followed by Tukey’s multiple comparison test (for equal variance) or Dunnett’s multiple comparison test (for unequal variance) (**a**–**e**). *P < 0.05, **P < 0.01. Exact *P* values: NC versus HFD 0.0003 (PD-L1^+^SPiDER^+^ cells) and <0.0001 (SPiDER^+^ cells), HFD versus HFD + Cana 0.0396 (PD-L1^+^SPiDER^+^ cells) and 0.0127 (SPiDER^+^ cells) (**a**); NC versus HFD 0.0002 (Macrophage), 0.048 (NK cell), 0.0267 (CD8^+^ T cell) and 0.0034 (CD69^+^CD8^+^ T cell), HFD versus HFD + Cana 0.0022 (macrophage), 0.0098 (NK cell), 0.0164 (CD8^+^ T cell) and 0.0177 (CD69^+^CD8^+^ T cell) (**b**); NC versus HFD 0.0082 (PD-L1^+^SPiDER^+^ cells) and <0.0001 (SPiDER^+^ cells), HFD versus HFD + AICAR 0.0463 (PD-L1^+^SPiDER^+^ cells) and 0.001 (SPiDER^+^ cells) (**c**); HFD versus HFD + Cana 0.0029 (PD-L1^+^SPiDER^+^ cells) and <0.0001 (SPiDER^+^ cells), HFD + Cana versus HFD + Cana + Comp. C 0.0011 (PD-L1^+^SPiDER^+^ cells) and <0.0001 (SPiDER^+^ cells) (**d**); sh-Cont versus Cana + sh-Cont 0.0291 (relative tdTomato intensity) and 0.0444 (tdTomato+ senescent cells count), Cana + sh-Cont versus Cana +sh-*Prkaa1* 0.0273 (relative tdTomato intensity) and 0.0269 (tdTomato+ senescent cells count) (**e**). Data are shown as the mean ± s.e. in plots of all individual data (**a**–**e**). Gating strategy in FACS analysis was shown in Supplementary Fig. 3a (**a**,**c**,**d**), 3b (**b**) and 3c (**e**). Source data

**Extended Data Fig. 6:**
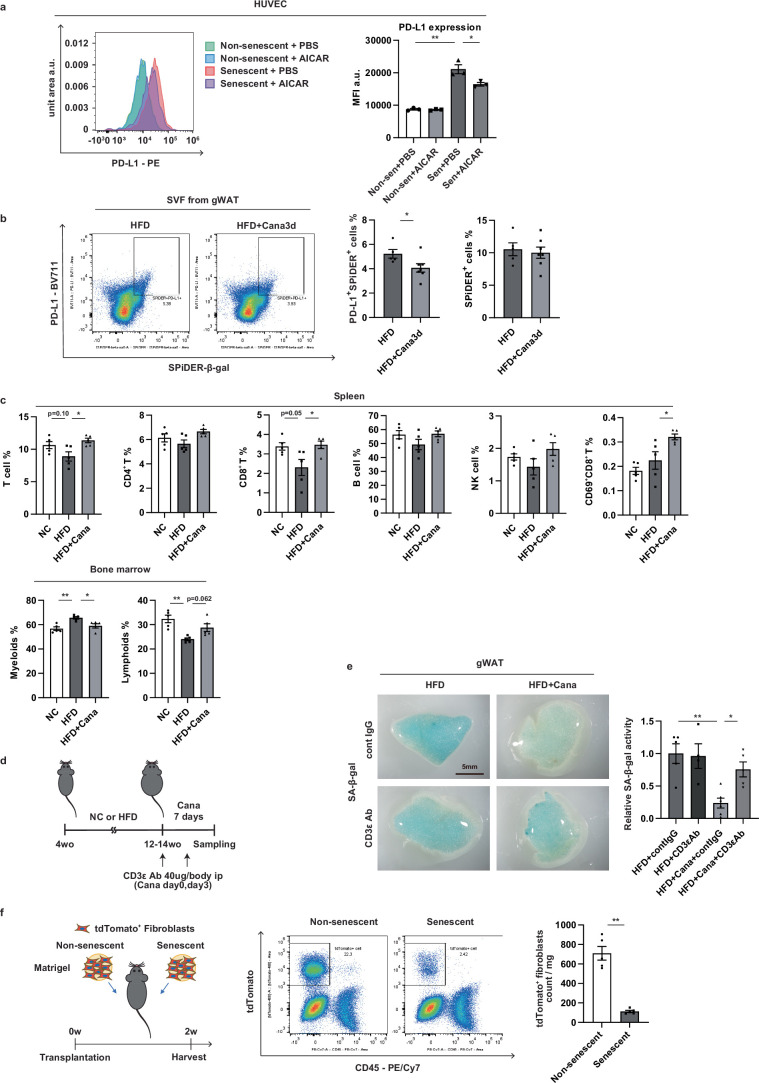
SGLT2 inhibition affects PD-L1 expression by senescent cells. **a**, FACS analysis for PD-L1 expression in irradiation-induced senescent HUVEC after 6 hours of AICAR treatment (n = 3 each). **b**, FACS analysis for PD-L1+ SPiDERβ-gal+ cells in stromal vascular fraction (SVF) obtained from gonadal white adipose tissue (gWAT) of high-fat diet (HFD) with or without canagliflozin (Cana) (n = 5, 7) on day 3. **c**, FACS analysis for immune cells in spleen and bone marrow of mice as prepared in Fig. 3a (n = 5 each). **d**, Protocol of the experiments to test the effects of CD3-neutralizung antibody on the senolytic effects of canagliflozin. NC, normal chow; HFD, high-fat diet. **e**, SA-β-gal activity in gWAT of mice as prepared in Extended Data Fig. 6d (n = 5, 4, 6, 5). **f**, FACS analysis of Matrigel transplantation model containing senescent fibroblasts (IR+) or non-senescent fibroblasts (IR–) (n = 5 each). IR, irradiation. Data were analyzed by two-way ANOVA followed by Tukey’s multiple comparison test (a, c, e) or two-tailed unpaired Student’s *t*-test (b, f). *P < 0.05, **P < 0.01. Exact P-value: Non-senescent+PBS versus Senescent+PBS: 0.0001, Senescent+PBS versus Senescent+AICAR: 0.0223 (a); HFD versus HFD+Cana3d: 0.0462 (PD-L1+ SPiDER+ cell) (b); NC versus HFD: 0.1075 (Spleen-T cell), 0.0503 (Spleen-CD8+ T cell), 0.0039 (BM-Myeloid) and 0.002 (BM-Lymphoid), HFD versus HFD+Cana: 0.0219 (T cell), 0.0345 (CD8+ T cell), 0.0328 (CD69+CD8+ T cell), 0.0433 (BM-Myeloid) and 0.062 (BM-Lymphoid) (c); HFD+contIgG versus HFD+Cana+contIgG: 0.0024, HFD+Cana+contIgG versus HFD+Cana+CD3εAb: 0.0405 (e); and Non-senescent versus Senescent: < 0.0001 (f). Data are shown as the mean ± SE in plots of all individual data (a–c, e, f). Gating strategy in FACS analysis was shown in Supplementary Fig. 3a (b), d (f), e (a), f (c), and g (c). Source data

Because AMPK activity has been shown to negatively regulate PD-L1 expression^27,28^, we decided to investigate the effect of SGLT2 inhibition on PD-L1. Consistent with previous reports, we found that AMPK activation by treatment with AICAR downregulated expression of PD-L1 in senescent human endothelial cells (Extended Data Fig. 6a). We then tested whether SGLT2 inhibition affected PD-L1 in HFD-fed mice. We found that the number of PD-L1-positive senescent cells was increased in the gWAT of HFD-fed mice compared with the NC group and that this increase was significantly reduced by short-term SGLT2 inhibitor treatment (Fig. 3a and Extended Data Fig. 6b). Consistent with these results, the numbers of natural killer (NK) cells and CD8^+^ T cells were reduced in the gWAT and other tissues (spleen and bone marrow) of mice fed HFD compared with the NC group, but short-term treatment with canagliflozin increased the numbers of these cells (Fig. 3b and Extended Data Fig. 6c). Similarly, treatment of HFD-fed mice with AICAR for a short time significantly reduced the number of PD-L1-positive senescent cells, which had increased because of the HFD (Fig. 3c). Treatment of HFD-fed mice with canagliflozin reduced the number of PD-L1-positive senescent cells compared with the control HFD group, but this reduction was inhibited by the AMPK inhibitor Compound C (Fig. 3d).

We next examined whether suppression of specific immune cells would inhibit the effects of canagliflozin on senescent cells. Specifically, we tested how administration of CD3-neutralizing antibody to HFD-fed mice affects the senolytic effects of canagliflozin (Extended Data Fig. 6d). The results showed that administration of CD3-neutralizing antibody attenuated the effects of canagliflozin on senescent cells (Extended Data Fig. 6e), suggesting that the senolytic effects of canagliflozin are at least partly due to activation of T cells.

To further investigate the effect of SGLT2 inhibition on senescent cell removal, we created a model in which fibroblasts derived from CAG-tdTomato reporter mice were irradiated to induce cellular senescence, mixed with Matrigel and implanted into subcutaneous tissue of wild-type mice. As a control, nonsenescent tdTomato-positive fibroblasts mixed with Matrigel were also transplanted into the subcutaneous tissue of the same mice. The results of fluorescence-activated cell sorting (FACS) analysis for tdTomato showed that senescent cells in the Matrigel were specifically eliminated, whereas nonsenescent cells were not (Extended Data Fig. 6f).

We next used this model to monitor the effect of the removal of senescent cells from Matrigel to determine the effect of canagliflozin and/or AMPK deletion on senescent cell removal. Mice were fed HFD for 8–10 weeks. tdTomato-positive senescent cells infected with either the sh-*Prkaa1* (a gene encoding AMPK catalytic subunit) vector or the sh-Scramble (control) vector were then transplanted into the subcutaneous tissue of the same mouse. After a short period (1 week) of treatment with canagliflozin, Matrigel was collected and examined using in vivo fluorescence imaging analysis and FACS analysis to determine the removal efficiency of senescent cells. The results showed that SGLT2 inhibition significantly reduced tdTomato-positive senescent cells and that this effect was abolished by knockdown of AMPK in senescent cells (Fig. 3e).

### Effects of SGLT2 inhibitor on aging phenotypes

**Fig. 4:**
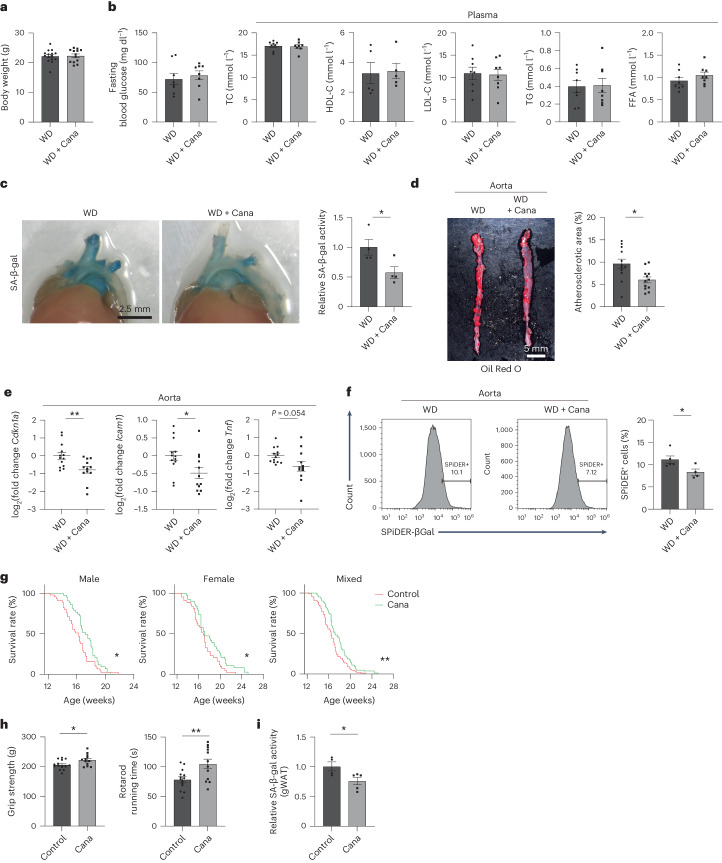
Effects of SGLT2 inhibitor on aging phenotypes. **a**,**b**, ApoE-KO mice were fed WD for 12 weeks and then treated with canagliflozin (Cana) for 2 weeks (WD + Cana). Body weight (*n* = 15, 13) (**a**), fasting blood glucose level and the lipid profile (*n* = 8 each) (**b**) were examined. **c**, SA-β-gal activity in the aorta of mice as prepared in **a** (*n* = 4 each). **d**, Oil Red O staining in the aorta of mice as prepared in **a** (*n* = 12 each). **e**, qPCR analysis for *Cdkn1a*, *Icam1* and *Tnf* in the aorta of mice as prepared in **a** (*n* = 13, 12). **f**, FACS analysis for SPiDER-βGal^+^ cells in the aorta of mice as prepared in **a** (*n* = 5, 4). **g**, Lifespan of Zmpste24 KO mice treated with canagliflozin (Cana) or vehicle (Cont) from 12 weeks of age (*n* = 46, 44 for males; *n* = 44, 38 for females). **h**, Middle-aged mice were treated with Cana or vehicle (Cont) from the age of 50 weeks and examined for physical activity at 70 weeks of age (*n* = 14, 12). **i**, SA-β-gal activity in gWAT of mice as prepared in **h** (*n* = 4, 5). Data were analyzed by two-tailed unpaired Student’s *t*-test (**a**–**f**,**h**,**i**). *P < 0.05, **P < 0.01. Exact *P* values: WD versus WD + Cana 0.0417 (**c**); WD versus WD + Cana 0.0085 (**d**); WD versus WD + Cana 0.0071 (*Cdkn1a*), 0.0258 (*Icam1*) and 0.0536 (*Tnf*) (**e**); WD versus WD + Cana 0.0438 (**f**); Cont versus Cana 0.0244 (male), 0.0446 (female) and 0.0038 (all) (**g**); Cont versus Cana 0.0279 (grip strength) and 0.0074 (rotarod running time) (**h**); Cont versus Cana 0.0408 (**i**). Data are shown as the mean ± s.e. in plots of all individual data (**a**–**f**,**h**,**i**). Survival curves were generated using the Kaplan–Meier method and compared with the log-rank test. In all analyses, *P* < 0.05 was considered to indicate statistical significance (**g**). Gating strategy in FACS analysis was shown in Supplementary Fig. 3d (**f**). FFA, free fatty acid; HDL-C, high-density lipoprotein cholesterol; LDL-C, low-density lipoprotein cholesterol; TC, total cholesterol; TG, triglyceride. Source data

Next, we examined whether inhibition of SGLT2 with canagliflozin was effective at reducing senescent cells in atherosclerotic plaques. Apolipoprotein E-knockout (ApoE-KO) mice were fed a western diet (WD) for 12 weeks and then treated with canagliflozin for 2 weeks. Canagliflozin had no effect on body weight, glucose levels or lipid profile, including plasma levels of cholesterol, triglycerides and free fatty acids, compared with the control group (Fig. 4a,b). However, there was significant improvement in senescence-like changes in the aorta, such as decreased SA-β-gal activity and *Cdkn1a* expression along with decreased plaque area, compared with the control group (Fig. 4c–e). Canagliflozin treatment also reduced inflammatory marker expression (Fig. 4e). In addition, FACS analysis showed a decrease in fluorescent β-gal-positive cells after canagliflozin treatment, suggesting that inhibition of SGLT2 could eliminate senescent cells from atherosclerotic plaques (Fig. 4f).

Hutchinson–Gilford progeria syndrome (HGPS) is a disease in which mutations of the LMNA gene cause premature aging, and there is evidence that senescence of adult somatic cells and stem cells has a crucial role in the premature aging of patients with HGPS^29^. Zinc metalloproteinase Ste24 homolog (Zmpste24) is an endoprotease with a critical role in the maturation of lamin A protein, and Zmpste24 KO mice exhibit growth retardation, alopecia, muscle weakness, osteoporosis and premature death, all of which are features of HGPS. To investigate whether inhibition of SGLT2 with canagliflozin could delay the development of premature aging, we treated Zmpste24 KO mice with canagliflozin or vehicle from the age of 12 weeks and assessed survival. The results showed that SGLT2 inhibition significantly prolonged the lifespan of these mice in both sexes compared with the control group (Fig. 4g). We also treated middle-aged wild-type mice (50 weeks old) with canagliflozin for 20 weeks and examined pathological aging phenotypes. The results showed that SGLT2 inhibition significantly improved physical activity, such as grip strength and rotarod running time, and decreased SA-β-gal activity in gWAT compared with the control group (Fig. 4h,i).

## Discussion

The accumulation of senescent cells in tissues is accelerated by DNA damage caused by aging or metabolic stress. Aging and pathological conditions such as diabetes and atherosclerosis are believed to impair endogenous senolysis mechanisms (anergy), which further accelerates the accumulation of senescent cells; however, the mechanism is unclear. In this study, we successfully developed a therapeutic strategy that targets anergy of senescent cells.

SGLT2 is a glucose and sodium transporter localized in the proximal tubule. Inhibition of SGLT2 lowers blood glucose by promoting the excretion of glucose into the urine, so SGLT2 was initially marketed as a treatment for diabetes. Subsequently, clinical studies demonstrated its efficacy in heart failure and chronic kidney disease^30–32^, and the indication was expanded; however, the mechanism of efficacy of SGLT2 inhibitors remains unclear. We suggest that the mechanism of action may include senolytic effects because accelerated accumulation of senescent cells in tissues has been reported in diabetes, heart failure and chronic kidney disease.

Because SGLT2 inhibition promotes excretion of glucose into the urine, it may induce a state of systemic metabolism similar to that of caloric restriction and fasting. Our metabolome analysis showed elevations in AICAR, which activates AMPK. We found that short-term administration of AICAR had a senolytic effect similar to that of the SGLT2 inhibitor canagliflozin, that inhibition of AMPK attenuated the senolytic effect of canagliflozin and that knockdown of AMPK in senescent cells attenuated the senolytic effect of SGLT2 inhibition, suggesting that AMPK activity mediates at least part of the senolytic effect of SGLT2 inhibitors. These results suggest that drugs that activate AMPK, such as biguanides, may have similar senolytic effects. By contrast, blood glucose-lowering treatment with insulin may act in a pro-senescence manner by activating the Akt-mammalian/mechanistic target of rapamycin pathway^33^. In fact, short-term insulin treatment showed an increasing trend in the expression of cellular senescence markers and SASP factors, even though blood glucose was reduced to the same level as that of SGLT2 inhibitors.

It is important to note that additional effects of canagliflozin have been observed on immune cells, including a direct inhibitory effect on T cell activation in culture^34^. However, our pharmacokinetic analysis of canagliflozin availability in plasma (Supplementary Fig. 2) indicates that the concentration shown to be inhibitory in vitro (10 µM) is considerably higher than the concentration of free-form canagliflozin (Supplementary note 1).

PD-L1 is known to play an important role as an immune checkpoint molecule in cancer^25^. A recent study showed that PD-L1 is expressed in some senescent cells and is also involved in anergy of senescent cells^26^. PD-1/PD-L1 inhibition has been shown to ameliorate various pathological aging traits by activating endogenous senolysis mechanisms^26^. Furthermore, activation of AMPK was found to negatively regulate PD-L1 expression by promoting degradation^27,28^, which is consistent with the results of our study. However, AMPK has been reported to regulate T cell activation^35^, and it is possible that SGLT2 inhibition directly affects T cell activity. Furthermore, multiple molecular mechanisms may exist that induce anergy of senescent cells, as well as cancer immunity^36,37^, and the possibility that SGLT2 inhibition regulates these anergy-related molecules cannot be ruled out. The identification of anergy-associated molecules and the elucidation of the mechanisms by which anergy is induced in senescent cells are expected to lead to the development of novel therapies targeting these molecules.

### Limitation

Most experiments were performed with male mice only, so possible sex differences in effects are not clear.

## Methods

### Animal models

All of the animal experiments were conducted in compliance with the protocol reviewed by the Institutional Animal Care and Use Committee of Niigata University and/or Juntendo University and were approved by the respective president. C57BL/6 mice were purchased from SLC Japan. Mice were maintained in a specific pathogen-free facility at 20–26 °C, 40–60% humidity, under a 12-h light and 12-h dark regimen. Mice were imposed on HFD (HFD32, CLEA Japan) or NC (CE-2, CLEA Japan or CRF-1, Oriental Yeast Japan) from 4 to 16 weeks old, unless otherwise described in the figure captions. ApoE-KO mice (C57BL/6 background) were obtained from the Jackson Laboratory and fed WD (F2HFD1, Oriental Yeast Japan) from age 4 to 18 weeks. We used Zmpste24-deficient mice (a model for HGPS) as a premature aging mouse model. Zmpste24 KO mice (MGI: 2158363) were obtained from S. G. Young^38^ and back-crossed to C57BL/6 background. p19^Arf^-DTR-luciferase mice (C57BL/6 background) and CAG-tdTomato mice (C57BL/6 background) were provided by M. Sugimoto^39^ and M. Abe^40^, respectively. To eliminate p19^Arf^-positive cells, DT (50 μg per kg body weight) was administered intraperitoneally every 2 weeks from age 4 weeks for 2 months. Canagliflozin was obtained from Mitsubishi Tanabe Pharma Corporation and administered by mixing into diets at 0.03% w/w from 3 days to 20 weeks. AICAR (Wako, cat. no. 015-22531) was administered intraperitoneally at a dose of 5 g per kg body weight in phosphate-buffered saline (PBS) once daily for 7 days; Compound C (dorsomorphin dihydrochloride; Wako, cat. no. 047-33763) was administered intraperitoneally at a dose of 10 mg per kg body weight in PBS once daily for 7 days; and insulin detemir was administered subcutaneously at a dose of 5 U per kg body weight in PBS once daily for the indicated period. For T cell depletion, Ultra-LEAF Armenian hamster anti-CD3ε antibody (BioLegend, cat. no. 100360) or Ultra-LEAF Armenian hamster isotype control immunoglobulin (Ig) G (BioLegend, cat. no. 400960) was administered intraperitoneally at a dose of 40 μg per body on day 0 and day 3 during canagliflozin treatment. All experiments except for Zmpste24 KO mice were analyzed in male mice.

### Histological analyses

SA-β-gal activity was examined using the following protocol. Freshly isolated tissue was incubated for 2 h at 37 °C in β-gal staining solution containing 1 mg ml^−^^1^ 5-bromo-4-chloro-3-indolyl-beta-d-galactoside (Takara, cat. no. 9031), 5 mmol l^−^^1^ potassium ferrocyanide, 5 mmol l^−1^ potassium ferricyanide, 150 mmol l^−^^1^ NaCl, 2 mmol l^−^^1^ MgCl_2_, 0.01% sodium deoxycholate and 0.02% Nonidet P-40, after which SA-β-gal activity was assessed by photographing the stained organs to measure the density of cyan color per area^5^. Atherosclerotic plaques were examined with Oil Red O staining. Whole aortas were dissected to remove adventitial fat, opened, pinned flat and fixed in 4% paraformaldehyde for 12 h at room temperature. The pinned aortas were then washed for 1 min with 60% isopropyl alcohol and incubated in 0.5% Oil Red O solution (Sigma-Aldrich, cat. no. O9755) in 60% isopropyl alcohol for 15 min at 37 °C for staining. Subsequently, the samples were briefly immersed in 60% isopropyl alcohol solution and then washed with double-distilled water. The Oil Red O-stained specimens were then photographed^6^. For histological sections, white adipose tissue samples were harvested, fixed in 10% formalin overnight, embedded in paraffin, sectioned and stained with hematoxylin and eosin. Reactive oxygen species were evaluated with dihydroethidium. For p53 detection, the following antibodies were used: anti-p53 antibody 1:25 (Leica Biosystems, cat. no. p53-CM5p-L), Biotin-SP (long spacer) AffiniPure donkey anti-rabbit IgG (H+L) 1:25 (Jackson ImmunoResearch, cat. no. 711-065-152), Streptavidin-Cy5 1:25 (Vector Laboratories, cat. no. SA-1500-1), wheatgerm agglutinin 1:50 (Invitrogen, cat. no. W11261) and Hoechst33258 1:1,000 (Invitrogen, cat. no. H35691000). Five fields per section were randomly selected and examined by confocal microscopy (Nikon AX). SA-β-gal activity, plaque area, crown-like structures, reactive oxygen species levels and p53-positive cells were quantified with ImageJ (v.1.53a).

### Physiological analyses

Forelimb grip strength (*g*) was measured using a grip strength meter (BIOSEB, cat. no. BIO-GS3). For each mouse, results were averaged from three trials. To measure endurance capacity, a rotarod performance test was performed with an accelerating rotarod machine (O’Hara & Co., cat. no. RRAC-3002). For this test, the mouse was placed on the lane rotating at 5–50 rpm, and the time latency (in seconds) until the mouse fell off the lane was recorded. For in vivo metabolic measurements, mice were housed individually to enable monitoring of body weight and food intake. Oxygen consumption was measured with an O_2_/CO_2_ metabolic measurement system (Columbus Instruments) according to the manufacturer’s instructions.

### Systemic metabolic parameters

Before the assay, mice were housed individually for 1 week. On the day of the glucose tolerance test (GTT), the mice were fasted for 6 h and then glucose was injected intraperitoneally at a dose of 1 g per kg body weight in the early afternoon. For the insulin tolerance test (ITT), mice were given human insulin intraperitoneally (1 U per kg body weight). Tail vein blood was collected at 0, 15, 30, 60 and 120 min after administration, and blood glucose levels were measured with a glucose analyzer (Sanwa Kagaku Kenkyusho).

### Cell isolation from mouse tissue

Tissues (visceral adipose tissue, aorta, Matrigel, ear skin, spleen and bone marrow) were excised, minced and digested with digesting solution (2 mg ml^−1^ collagenase type II (Worthington, cat. no. CLS2) and 1 mM calcium chloride (CaCl_2_) in PBS) for 20–30 min at 37 °C except spleen and bone marrow. Lysate was subsequently filtered through a nylon mesh (40 μm) and red blood cell lysis was achieved with an ammonium chloride-based lysing buffer (Pharm Lyse; BD, cat. no. 555899). Cells were resuspended in PBS supplemented with 1% FBS (Gibco, cat. no. 10437028) and 5 mM EDTA for FACS analysis or in DMEM (Sigma, cat. no. D6046) containing 10% FBS and 1% penicillin/streptomycin (P/S; Gibco, cat. no. 15140122) for cell culture.

### Cell culture

Human umbilical vein endothelial cells (HUVEC) were purchased from Lonza and cultured according to the manufacturer’s instructions. Human fetal lung fibroblasts (IMR90) were purchased from KAC Co (cat. no. EC85020204-F0). Mouse ear fibroblasts were obtained from the ears of 4-week-old wild-type or CAG-tdTomato mice. IMR90 and mouse ear fibroblasts were cultured in DMEM (Sigma, cat. no. D6046) containing 10% FBS and 1% P/S. To induce cellular senescence of ear fibroblasts, IMR90 and HUVEC, ionizing irradiation was performed at 10 Gy. Senescent fibroblasts and HUVEC were subjected to further experiments or analysis at 7–10 days after irradiation. In some experiments, cells undergoing replicative senescence were used. We defined replicative senescent cells as the cultures that do not increase in the cell number and remain sub-confluent for 2 weeks.

### Adeno-associated viral vector

Adeno-associated virus (serotype: AAV-DJ) with U6 promoter-mediated short hairpin RNA construct was purchased from VectorBuilder. Sequences of sh-Scramble and sh-Prkaa1 were as follows:

sh-Scramble: CCTAAGGTTAAGTCGCCCTCGCTCGAGCGAGGGCGACTTAACCTTAGG

sh-Prkaa1: GAATCCTCATAGACCTTATTACTCGAGTAATAAGGTCTATGAGGATTC.

### Matrigel transplantation

Irradiated mouse ear fibroblasts from CAG-tdTomato mice infected with AAV-sh-Scramble or AAV-sh-Prkaa1 (10^5^ multiplicity of infection) were mixed with Matrigel (Corning, cat. no. 35237) at 5 × 10^5^ ml^−1^ and subcutaneously transplanted into the back of anesthetized wild-type mice. Two weeks after transplantation, Matrigel was collected and used for in vivo fluorescence imaging analysis and FACS analysis.

#### In vivo fluorescence imaging analysis

In vivo fluorescence imaging analysis was performed with an in vivo imaging system (Perkin Elmer). Mice were shaved ventrally, anesthetized with isoflurane (Wako, cat. no. 099-06571) and injected intraperitoneally with luciferin (Promega, cat. no. P1043; 150 mg per kg body weight) according to the manufacturer’s instructions. Luciferase activity was monitored starting 5 min after luciferin injection. tdTomato fluorescence was measured by transferring harvested Matrigel to each well of a 96-well clear-bottom plate filled with PBS. To quantify luciferase activity or tdTomato fluorescence, signals were analyzed with Living Image software (v.4.5.5; Perkin Elmer).

#### RNA analysis

Total RNA (1 μg) was isolated from tissue samples with QIAZOL (QIAGEN). Real-time quantitative polymerase chain reaction (qPCR) was performed with a Light Cycler 480 (Roche) with the Universal Probe Library and the Light Cycler 480 Probes Master (Roche) or SYBR Green and QUantiStudio6 Pro (Applied Biosystems) according to the manufacturer’s instructions. The primers and their sequences are listed below (Rplp0 was used as the internal control).

Mouse primers (forward, backward):

*Cdkn1a*; 5′-TCCACAGCGATATCCAGACA-3′, 5′-GGACATCACCAGGATTGGAC-3′

*Cdkn2a*; 5′-GGGTTTTCTTGGTGAAGTTCG-3′, 5′-TTGCCCATCATCATCACCT-3′

*Ccl2*; 5′-CATCCACGTGTTGGCTCA-3′, 5′-GATCATCTTGCTGGTGAATGAGT-3′

*Tnf*; 5′-TCTTCTCATTCCTGCTTGTGG-3′, 5-′CTGTAGCCCACGTCGTAGC-3′

*Rplp0*; 5′-GATGCCCAGGGAAGACAG-3′, 5′-ACAATGAAGCATTTTGGATAA-3′.

For measurement of *Cdkn2d* messenger RNA expression, qPCR was performed with TaqMan FAM (Applied Biosystems, cat. no. 4331182) or VIC (Applied Biosystems, cat. no. 4448484) probe and predesigned *Cdkn2d* primer (Applied Biosystems, cat. no. Mm00486943_m1) or *Rplp0* primer (Applied Biosystems, cat. no. Mm00725448_s1) and QUantiStudio6 Pro.

#### RNA sequencing analysis

Total RNA was extracted from mouse gWAT using the Maxwell RSC 48 Instrument (Promega) and Maxwell RSC simplyRNA Tissue Kit (Promega, cat. no. AS1340) in accordance with the manufacturer’s instructions. The complementary DNA libraries were generated with the TruSeq Stranded mRNA Library Prep Kit (Illumina). The quality of total RNA and cDNA was assessed with an Agilent 2100 Bioanalyzer with the RNA6000 nano kit and DNA7500 kit (Agilent Technologies). Sequencing was performed with the NovaSeq6000 system (Illumina) with a pair-end-read sequencing length of 150 bp. Acquired read data were processed and analyzed by nf-core/rnaseq pipeline (v.3.12.0) as the default setting on nextflow software (v.23.10.0). The *z*-score was calculated from transcript per million data, and its heatmap was generated in RStudio (v.2023.12.0+369) and R (v.4.3.2).

#### Western blot analysis

Whole-cell lysates were prepared in lysis buffer (10 mmol l^−1^ Tris–HCl, pH 8, 140 mmol l^−^^1^ NaCl, 5 mmol l^−^^1^ EDTA, 0.025% NaN3, 1% Triton X-100, 1% deoxycholate, 0.1% SDS, 1 mmol l^−^^1^ phenylmethyl sulfonyl fluoride, 5 μg ml^−^^1^ leupeptin, 2 μg ml^−^^1^ aprotinin, 50 mmol l^−^^1^ NaF, and 1 mmol l^−^^1^ Na_2_VO_3_). The lysates (20–50 μg) were then resolved by SDS–PAGE and proteins were transferred to a polyvinylidene difluoride membrane (Millipore) that was incubated with the primary antibody and then with anti-rabbit or anti-mouse IgG conjugated with horseradish peroxidase (Jackson). Specific proteins were detected by enhanced chemiluminescence (Cytiva, cat. no. RPN2106 or RPN2232). The primary antibodies for western blotting were anti-p53 antibody (Cell Signaling, cat. no. 2524 or Leica, cat. no. NCL-L-p53-CM5p), anti-phospho-AMPKα antibody (Cell Signaling, cat. no. 2535), anti-AMPKα antibody (Cell Signaling, cat. no. 5831) and anti-αTubulin antibody (Cell Signaling, cat. no. 2125). All primary antibodies were used at a dilution of 1:1,000, except for anti-αTubulin antibody, which was used at a dilution of 1:5,000. The secondary antibody, peroxidase-conjugated AffiniPure goat anti-rabbit IgG (H+L; Jackson ImmunoResearch, cat. no. 111-035-003) was used for all primary antibodies except anti-p53 antibody (1C12), for which peroxidase-conjugated AffiniPure goat anti-mouse IgG (light chain specific) (Jackson ImmunoResearch, cat. no. 115-035-174) was used. All secondary antibodies were used at a dilution of 1:5,000.

#### Enzyme-linked immunosorbent assay

The plasma concentration of mouse CCL2 and tumor necrosis factor was measured using an enzyme-linked immunosorbent assay kit (CCL2: Abcam, cat. no. ab208979; tumor necrosis factor: Abcam, cat. no. ab208348) according to the manufacturer’s instruction sheet. Briefly, 50 µl of mouse plasma was mixed with capture antibody and detection antibody in a clear, flat-bottom strip well and subsequently incubated for 1 h on an orbital shaker at room temperature. After the wells were washed with wash buffer, 50 µl of 3,3′,5,5′-tetramethylbenzidine substrate solution was incubated for 20 min, and the reaction was then stopped with 50 µl of stop solution. The concentration of each protein was calculated according to the optical density, which was acquired by measuring the absorbance at wavelengths of 450 and 570 nm.

#### FACS analysis

Isolated cells were stained with cell marker antibodies and 10 μM SPiDER-βGal (Dojindo, cat. no. SG02) for 30 min at room temperature. Cells from adipose tissue were incubated with Fcγ blocker 1:100 (BD, cat. no. 553141) for 5 min at room temperature before staining with cell marker antibodies. The following antibodies were used: BV421-conjugated anti-mouse CD31 1:100 (BioLegend, cat. no. 102424), Phycoerythrin(PE)/Cy7-conjugated anti-mouse CD45 1:100 (BioLegend, cat. no. 103114), BB515-conjugated anti-mouse Cd11b 1:100 (BD, cat. no. 564454), BB700-conjugated anti-mouse Cd11b 1:100 (BD, cat. no. 566416), PE-conjugated anti-mouse CD3ε 1:100 (BioLegend, cat. no. 100302), Allophycocyanin(APC)/Cy7-conjugated anti-mouse Nk1.1 1:100 (BioLegend, cat. no. 108724), BV650-conjugated anti-mouse CD19 1:100 (BioLegend, cat. no. 115541), BB700-conjugated anti-mouse CD4 1:100(BD, cat. no. 566407), Pacific Blue-conjugated anti-mouse CD8a 1:100(BioLegend, cat. no. 100725), BV711-conjugated anti-mouse CD69 antibody 1:100 (BioLegend, cat. no. 104537), BV711-conjugated anti-mouse CD274 (PD-L1) antibody 1:100 (BioLegend, cat. no. 124319) and PE-conjugated anti-human CD274 (PD-L1) antibody 1:100 (BioLegend, cat. no. 329706). Cells were washed and resuspended in FACS buffer and analyzed by a spectral cell analyzer (Sony, cat. no. ID7000) or a cell sorter (Sony, cat. no. SH800S). Data were collected and analyzed with ID7000 software (v.1.1.0.11041, Sony), SH800S cell sorter software (v.2.1.6, Sony) and FlowJo (v.10.8.1, BD). The gating strategies are shown in the Supplementary Fig. 3.

#### Cell viability analysis (MTT assay)

Cells were resuspended in a flat, clear-bottom 96-well plate at 5 × 10^3^ and treated with canagliflozin in 1% dimethylsulfoxide for 48 h. The medium was then replaced with 0.5 mg ml^−1^ of 3-(4,5-Dimethyl-2-thiazolyl)-2,5-diphenyltetrazolium Bromide (MTT) (TCI, cat. no. D0801). After 3 h incubation in a 5% CO_2_ incubator at 37°C, the medium was replaced with dimethylsulfoxide, and the absorbance was measured at a wavelength of 570 nm.

#### Metabolomic analysis

Metabolomic analyses were performed by capillary electrophoresis time-of-flight/mass spectrometry. Mouse blood samples were immediately centrifuged, and 40 μl of plasma was mixed with 360 μl of methanol containing l-methionine sulfone (Wako, cat. no. 502-76641), MES (Dojindo, cat. no. 349-01623) and CSA (Wako, cat. no. 037-01032) (all at 20 μM). The aqueous layer was then extracted with chloroform, filtered and subjected to capillary electrophoresis time-of-flight/mass spectrometry^41^.

#### Measurement of plasma canagliflozin concentration

Plasma concentrations of canagliflozin were determined by ultra-performance liquid chromatography/tandem-mass spectrometry (LC/MS/MS) at Mediford Corporation. For the measurement, 40 μl of mouse plasma was mixed with an internal standard (IS; [^14^C_6_] canagliflozin) and applied to an Oasis HLB µElution plate (Waters Corporation) for extraction. Canagliflozin and IS were eluted with acetonitrile and then analyzed by LC/MS/MS. Reverse-phase chromatography was performed on an ACQUITY ultra-performance liquid chromatograph (Waters Corporation) equipped with an Inertsil ODS-HL column (1.9 µm, 2.1 × 50 mm; GL Sciences) maintained at 40 °C. The analytes were eluted by linear gradient elution with 10 mmol l^−1^ ammonium acetate and acetonitrile/100 mmol l^−1^ ammonium acetate (90:10, v/v) at a flow rate of 0.5 ml min^−1^. Mass spectrometric detection was performed with a Triple Quad 5500+ mass spectrometer (SCIEX) equipped with a Turbo Spray source operating in positive mode. Canagliflozin and IS were monitored by a multiple reaction monitoring with monitor ions (*m*/*z*) of 462 > 249 and 468 < 255, respectively.

#### Measurement of protein binding rate of canagliflozin

In vitro protein binding of canagliflozin was examined at Mediford Corporation by the equilibrium dialysis method with two different media (EBM2 bullet kit containing 2% FBS, and DMEM containing 10% FBS). Then, 10 µmol l^−1^ canagliflozin was added to each medium, and the solution was incubated in a rapid equilibrium dialysis device for 24 h at 37 °C in a 5% CO_2_ incubator. After equilibrium dialysis, the concentrations of canagliflozin in the samples were determined by LC/MS/MS. The analysis samples were prepared by mixing PBS (dialysis sample), medium 1 and medium 2 at a ratio of 8:1:1 by volume.

### Statistics and reproducibility

Data were analyzed with GraphPad Prism 9 software (v.9.3.1, MDF). All data are from different biological replicates and shown as the mean ± s.e. Differences between two groups were examined by the two-tailed Student’s *t*-test. Two-way analysis of variance (ANOVA) tests followed by Tukey’s multiple comparison test (for equal variance) or Dunnett’s multiple comparison test (for unequal variance) were applied for three or more groups. Survival curves were calculated using the Kaplan–Meier method and were compared with the log-rank test. In GTT and ITT analyses, data were analyzed with repeated measures ANOVA, followed by Tukey’s multiple comparison test. In all analyses, a *P* value <0.05 was considered statistically significant.

Sample size was determined based on previously published experiments^7^ where statistic differences were observed. Outliers and abnormal values were excluded by Smirnov–Grubbs test with common threshold (*α* = 0.05) in case results follow a Gaussian distribution. All animals were randomly assigned to experimental and control groups by the investigator at baseline according to a prespecified number in each replicate, after which key covariates (such as body weight and blood glucose levels) were confirmed to be equivalent between groups. In cell culture experiments, all wells of cultured cells were prepared at the same cell density and sequentially assigned to the different treatment groups. Mouse experiments for physiological, metabolic and pathological analysis were blinded. Other experiments including in vitro and ex vivo studies, investigators were blinded to allocation during experiments and outcome assessments.

### Reporting summary

Further information on research design is available in the Nature Portfolio Reporting Summary linked to this article.

### Supplementary information

Supplementary Information Supplementary Figs. 1–3 and Note 1.

Reporting Summary

Supplementary Table 1 Metabolomic analysis.

### Source data

Source Data Fig. 1 Statistical source data.

Source Data Fig. 1 Unprocessed western blots.

Source Data Fig. 2 Statistical source data.

Source Data Fig. 2 Unprocessed western blots.

Source Data Fig. 3 Statistical source data.

Source Data Fig. 4 Statistical source data.

Source Data Extended Data Fig. 1 Statistical source data.

Source Data Extended Data Fig. 2 Statistical source data.

Source Data Extended Data Fig. 2f Unprocessed western blots.

Source Data Extended Data Fig. 3 Statistical source data.

Source Data Extended Data Fig. 3e Unprocessed western blots.

Source Data Extended Data Fig. 4 Statistical source data.

Source Data Extended Data Fig. 5 Statistical source data.

Source Data Extended Data Fig. 5b Unprocessed western blots.

Source Data Extended Data Fig. 6 Statistical source data.

## Data Availability

Gene expression data for RNA sequencing in mouse gWAT are available through the Gene Expression Omnibus database (GSE252539). Source data are provided with this paper. Other data supporting the conclusions of the study are available from the corresponding author upon request.
