# Supplementary material for: SGLT2 inhibition eliminates senescent cells and alleviates pathological aging
Source: Nat Aging. 2024 May 30;4(7):926–38. doi: 10.1038/s43587-024-00642-y (PMC11257941; doi:10.1038/s43587-024-00642-y)
Supplement: Supplementary file 1 — Supplementary Figs. 1–3 and Note 1. [file 43587_2024_642_MOESM1_ESM.pdf]

nature aging

Supplementary information https://doi.org/10.1038/s43587-024-00642-y

SGLT2 inhibition eliminates senescent cells and alleviates pathological aging

In the format provided by the authors and unedited

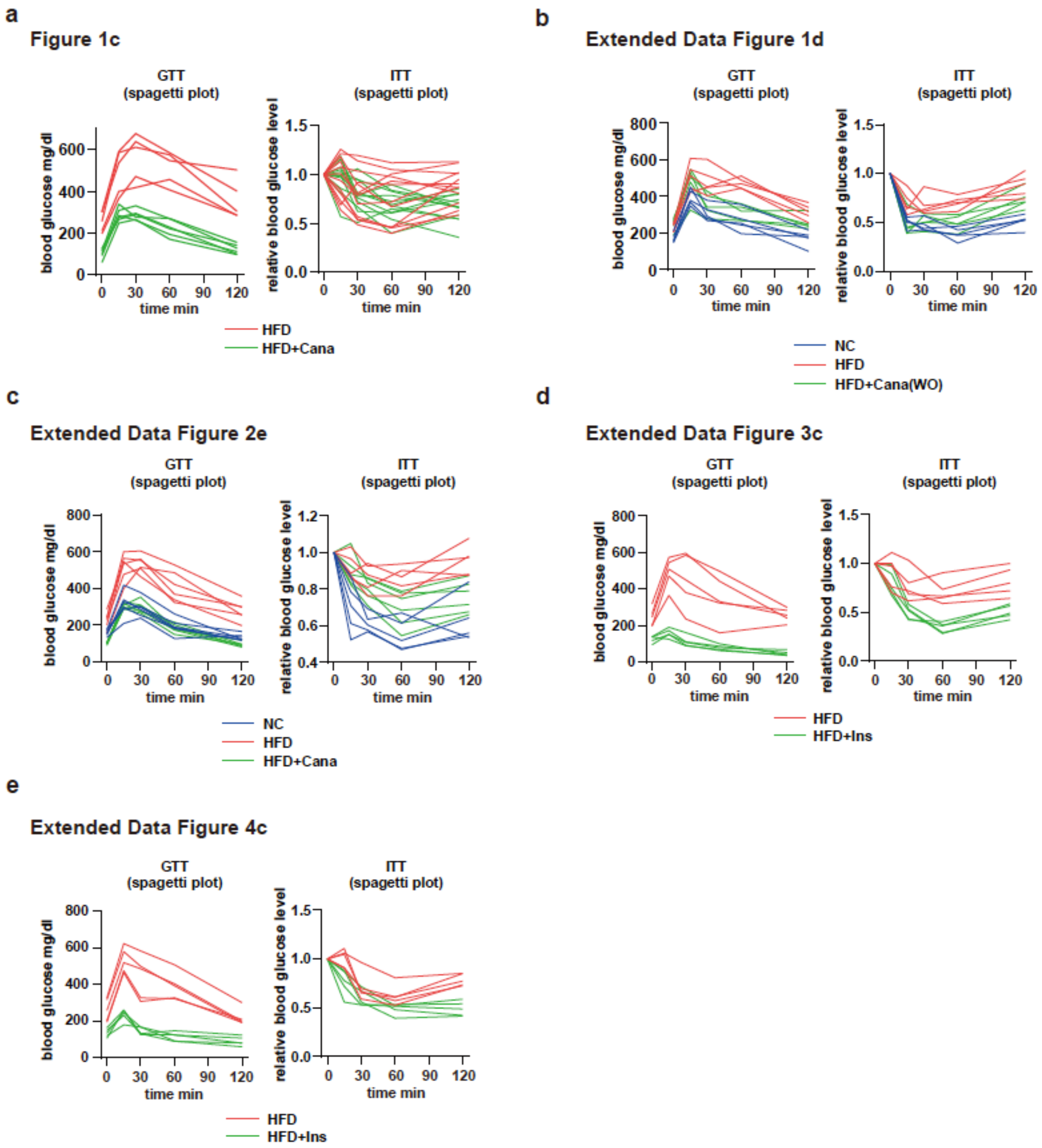

Supplementary Figure 1: The spaghetti plot of glucose tolerance test and insulin tolerance test.

a, Glucose tolerance test (GTT, n = 5, 6) and insulin tolerance test (ITT, n=12 each) in mice as prepared in Fig. 1c. **b,** GTT (n = 5, 6) and ITT (n = 12 each) in mice as prepared in Extended Data Fig. 1d. **c,** GTT (n = 6 each) and ITT (n = 5, 5, 6) in mice as prepared in Extended Data Fig. 2e. **d,** GTT and ITT in mice as prepared in Extended Data Fig. 3c (n = 6 each). **e,** GTT and ITT in mice as prepared in Extended Data Fig. 4c (n = 5 each).

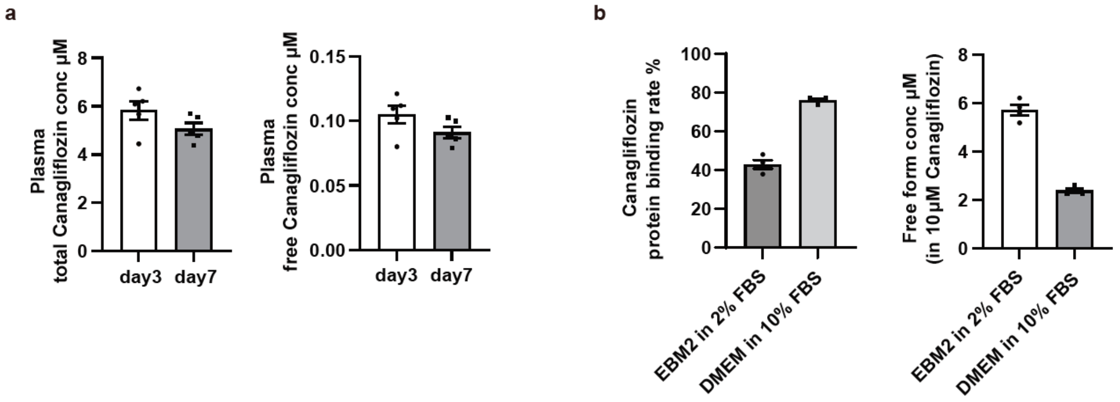

Supplementary Figure 2: Pharmacokinetics of canagliflozin.

a, 0.03 w/w% canagliflozin was administered to mice by mixed feeding, and plasma concentrations were measured on day 3 and day 7 (n = 5). Concentrations of free-form canagliflozin are shown in the right graph. **b,** The protein binding rate of canagliflozin was measured under 2% and 10% FBS culture conditions (n = 4). Concentrations of free-form canagliflozin are shown in the right graph. Data are shown as the mean ± SE in plots of all individual data

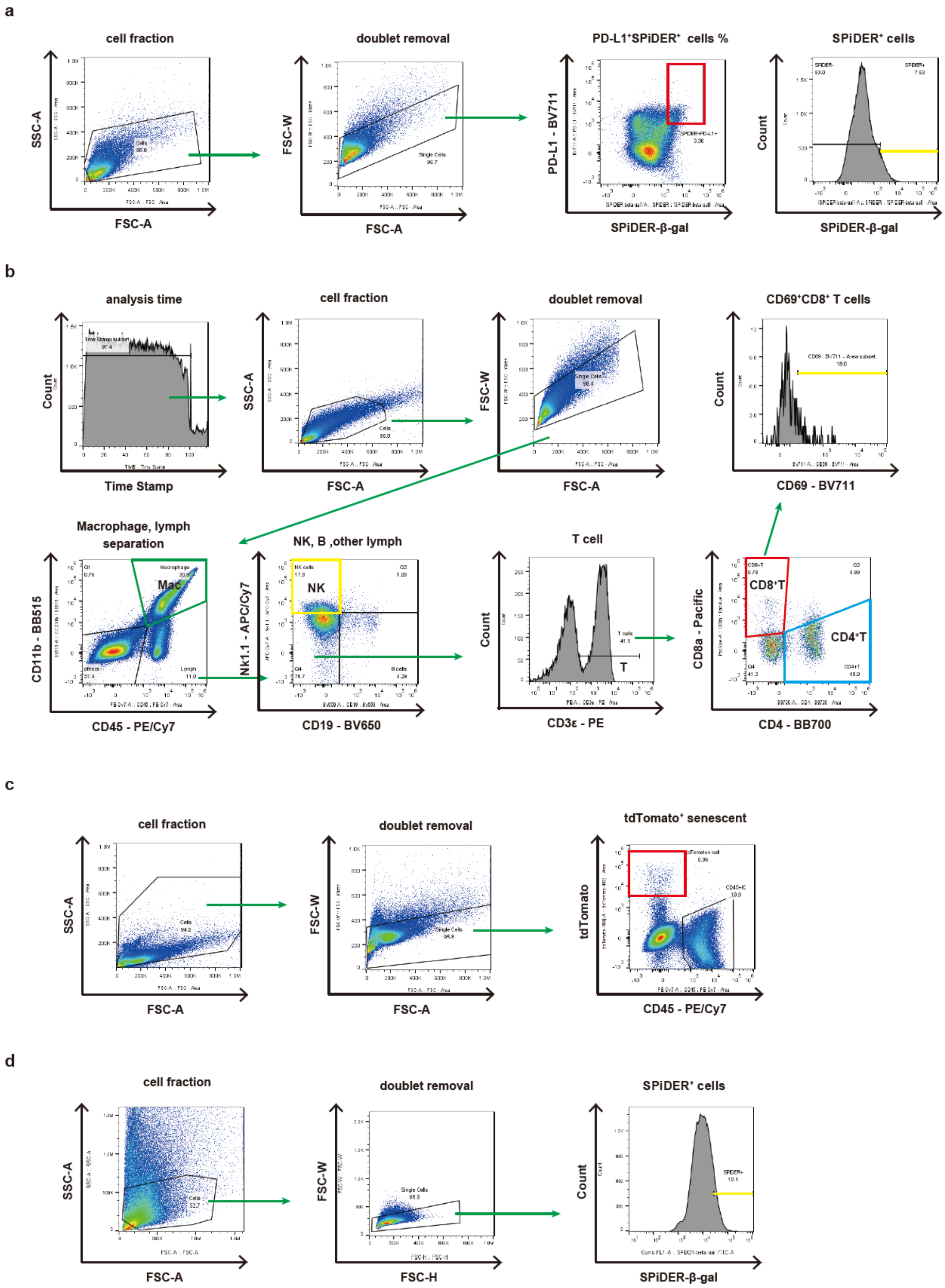
a
cell fraction
SSC-A
FSC-A
Cells
doublet removal
FSC-W
FSC-A
Single Cells
PD-L1⁺SPiDER⁺ cells %
PD-L1 - BV711
SPiDER-β-gal
SPiDER+PD-L1+
SPiDER⁺ cells
Count
SPiDER-β-gal
b
analysis time
Count
Time Stamp
cell fraction
SSC-A
FSC-A
doublet removal
FSC-W
FSC-A
CD69⁺CD8⁺ T cells
Count
CD69 - BV711
Macrophage, lymph separation
CD11b - BB515
CD45 - PE/Cy7
Mac
NK, B ,other lymph
Nk1.1 - APC/Cy7
CD19 - BV650
NK
T cell
Count
CD3ε - PE
T
CD8a - Pacific
CD4 - BB700
CD8⁺T
CD4⁺T
c
cell fraction
SSC-A
FSC-A
doublet removal
FSC-W
FSC-A
tdTomato⁺ senescent
tdTomato
CD45 - PE/Cy7
d
cell fraction
SSC-A
FSC-A
doublet removal
FSC-W
FSC-H
SPiDER⁺ cells
Count
SPiDER-β-gal

e

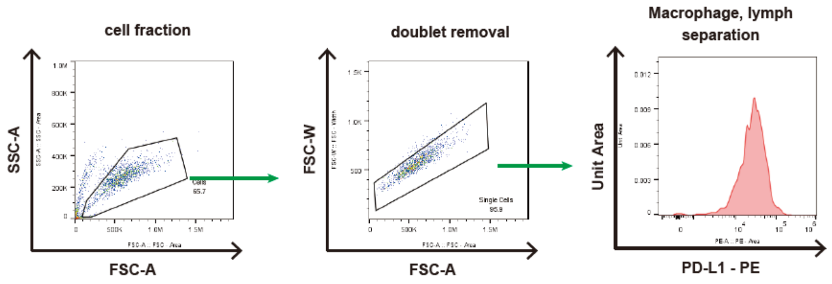
cell fraction
doublet removal
Macrophage, lymph separation
SSC-A
FSC-A
FSC-W
Unit Area
PD-L1 - PE

f

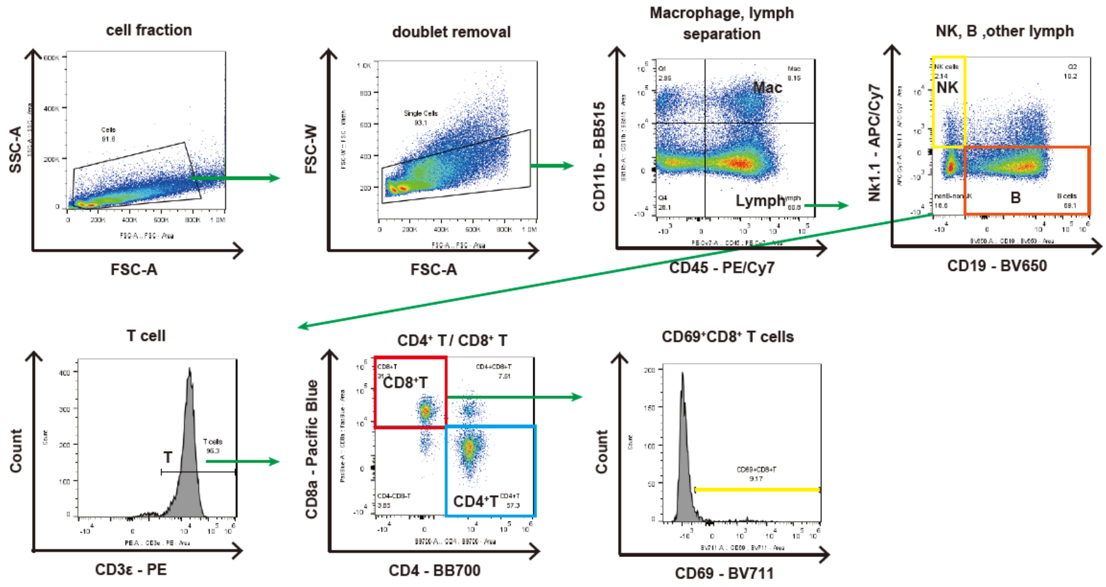
cell fraction
doublet removal
Macrophage, lymph separation
NK, B ,other lymph
SSC-A
FSC-A
FSC-W
CD11b - BB515
CD45 - PE/Cy7
Mac
Lymph
NK1.1 - APC/Cy7
CD19 - BV650
NK
B
T cell
CD4⁺ T / CD8⁺ T
CD69⁺CD8⁺ T cells
Count
CD3ε - PE
T
CD8a - Pacific Blue
CD4 - BB700
CD8⁺T
CD4⁺T
CD69 - BV711

g

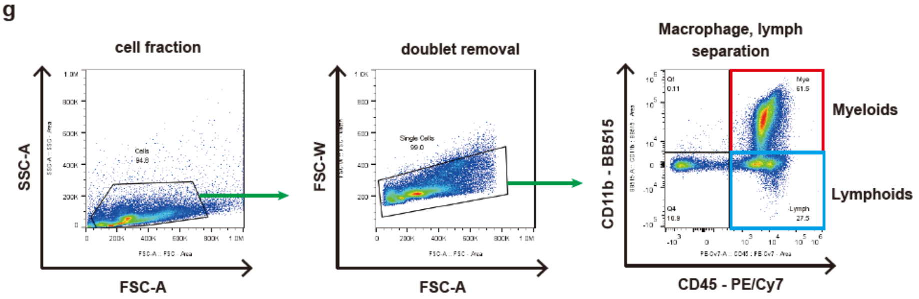
cell fraction
doublet removal
Macrophage, lymph separation
SSC-A
FSC-A
FSC-W
CD11b - BB515
CD45 - PE/Cy7
Myeloids
Lymphoids

Supplementary Figure 3 : Strategy for FACS analyses.

a, Gating strategy for Figure 3a, c, d, and Extended Data Figure 6b (gWAT). **b,** Gating strategy for Figure 3b (gWAT). **c,** Gating strategy for Figure 3e, and Extended Data Figure 6f (gWAT). **d,** Gating strategy for Figure 4f (Aorta), and Extended Data Figure 2d (gWAT). **e,** Gating strategy for Extended Data Figure 6a (HUVEC). **f,** Gating strategy for Extended Data Figure 6c (Spleen). **g,** Gating strategy for Extended Data Figure 6c (Bone marrow). Isolated cells from the SVF fraction in gWAT, aorta, spleen, or bone marrow were subjected to FACS analysis for SPiDER-β-gal or cell surface makers. The number of $SPiDER^+$ senescent cells, PD-$L1^+SPiDER^+$ cells, macrophages ($CD45^+$ $CD11b^+$), T cells ($CD45^+$ $CD11b^-$ $CD3\varepsilon^+$), $CD4^+$ T cells ($CD45^+$ $CD11b^-$ $CD3\varepsilon^+$ $CD4^+$), $CD8^+$ T cells ($CD45^+$ $CD11b^-$ $CD3\varepsilon^+$ $CD8a^+$), activated $CD8^+$ T cells ($CD45^+$ $CD11b^-$ $CD3\varepsilon^+$ $CD8a^+$ $CD69^+$), B cells ($CD45^+$ $CD11b^-$ $CD19^+$) and NK cells ($CD45^+$ $CD11b^-$ $NK1.1^+$) was examined. For bone marrow derived cells, the number of myeloid cells ($CD45^+$ $CD11b^+$), lymphoid cells ($CD45^+$ $CD11b^-$) was examined. In HUVECs, mean fluorescent intensity from PD-L1 antibody was measured.

Supplementary note 1

Although Jenkins et al. recently showed that canagliflozin has a direct inhibitory effect on T-cell activation in culture, the concentration they used (10 µM) was well above the physiological concentration (~1 µM), and its effect was not physiological. The rationale for assuming that the physiological concentration of canagliflozin is less than 1 µM is as follows:

- When canagliflozin (0.03 w/w%) is administered to mice by mixed feeding, the plasma concentration on day 7 is approximately 5.06 µM (Supplementary Figure 2a); however, the plasma concentration of the free form is approximately 91.1 nM because the plasma protein binding of canagliflozin is 98.2% (https://www.accessdata.fda.gov/drugsatfda_docs/nda/2013/204042Orig1s000TOC.cfm).
- When 100 mg of canagliflozin is administered repeatedly to patients with type 2 diabetes for 16 days, the maximum plasma concentration is 2.56 µM and the concentration of unbound canagliflozin is 43.4 nM (Iijima H et al. Adv Ther. 2015; 32: 768-82).
- In contrast, under 10% FBS culture conditions, the protein binding of canagliflozin is estimated to be approximately 76.1% (Supplementary Fig. 2b), resulting in a free plasma concentration of 2,390 nM at 10 µM, which is 25 to 50 times higher than the physiological concentration.
