# Supplementary material for: SGLT2 inhibition eliminates senescent cells and alleviates pathological aging
Source: Nat Aging. 2024 May 30;4(7):926–38. doi: 10.1038/s43587-024-00642-y (PMC11257941; doi:10.1038/s43587-024-00642-y)
Supplement: Supplementary file 2 — Reporting Summary [file 43587_2024_642_MOESM2_ESM.pdf]

Corresponding author(s): Tohru Minamino
Last updated by author(s): Apr 7, 2024

Reporting Summary

Nature Portfolio wishes to improve the reproducibility of the work that we publish. This form provides structure for consistency and transparency in reporting. For further information on Nature Portfolio policies, see our Editorial Policies and the Editorial Policy Checklist.

Statistics

For all statistical analyses, confirm that the following items are present in the figure legend, table legend, main text, or Methods section.

n/a	Confirmed	
☐	☒	The exact sample size (*n*) for each experimental group/condition, given as a discrete number and unit of measurement
☐	☒	A statement on whether measurements were taken from distinct samples or whether the same sample was measured repeatedly
☐	☒	The statistical test(s) used AND whether they are one- or two-sided<br>*Only common tests should be described solely by name; describe more complex techniques in the Methods section.*
☒	☐	A description of all covariates tested
☒	☐	A description of any assumptions or corrections, such as tests of normality and adjustment for multiple comparisons
☐	☒	A full description of the statistical parameters including central tendency (e.g. means) or other basic estimates (e.g. regression coefficient) AND variation (e.g. standard deviation) or associated estimates of uncertainty (e.g. confidence intervals)
☐	☒	For null hypothesis testing, the test statistic (e.g. *F*, *t*, *r*) with confidence intervals, effect sizes, degrees of freedom and *P* value noted<br>*Give P values as exact values whenever suitable.*
☒	☐	For Bayesian analysis, information on the choice of priors and Markov chain Monte Carlo settings
☒	☐	For hierarchical and complex designs, identification of the appropriate level for tests and full reporting of outcomes
☒	☐	Estimates of effect sizes (e.g. Cohen's *d*, Pearson's *r*), indicating how they were calculated

Our web collection on statistics for biologists contains articles on many of the points above.

Software and code

Policy information about availability of computer code

Data collection	ID7000 software (version 1.1.0.11041, Sony) for FACS data acquisiton<br>SH800S cell sorter software (version 2.1.6, Sony) for FACS data acquisiton<br>Living Image software (version 4.5.5, Perkin Elmer) for luminescence signal acquisiton.
Data analysis	nextflow software (version 23.10.0) for RNA-seq analysis<br>nf-core/rnaseq pipeline (version 3.12.0) for RNA-seq analysis<br>RStudio (version 2023.12.0+369) for heatmap creation from RNA-seq analysis data<br>R (version 4.3.2) for heatmap creation from RNA-seq analysis data<br>ImageJ (version 1.53a) for quantitative analysis of acquired pictures (Western blotting, SA-β-gal staining, HE staining and immuno-staining).<br>FlowJo (version 10.8.1, BD) for FACS analysis (gating)<br>Living Image software (version 4.5.5, Perkin Elmer) for IVIS data analysis<br>GraphPad Prism 9 software (version 9.3.1, MDF) for statistical analysis and graph creation.

For manuscripts utilizing custom algorithms or software that are central to the research but not yet described in published literature, software must be made available to editors and reviewers. We strongly encourage code deposition in a community repository (e.g. GitHub). See the Nature Portfolio guidelines for submitting code & software for further information.

Data

Policy information about availability of data

All manuscripts must include a data availability statement. This statement should provide the following information, where applicable:

- Accession codes, unique identifiers, or web links for publicly available datasets
- A description of any restrictions on data availability
- For clinical datasets or third party data, please ensure that the statement adheres to our policy

Gene expression data for RNA sequencing in mouse gWAT are available through the Gene Expression Omnibus database (GSE252539). Other data supporting the conclusions of the study are available as source data files, or are available from the corresponding author upon request.

Human research participants

Policy information about studies involving human research participants and Sex and Gender in Research.

Reporting on sex and gender	No human researchs were included in the study.
Population characteristics	N/A
Recruitment	N/A
Ethics oversight	N/A

Note that full information on the approval of the study protocol must also be provided in the manuscript.

Field-specific reporting

Please select the one below that is the best fit for your research. If you are not sure, read the appropriate sections before making your selection.

☒ Life sciences ☐ Behavioural & social sciences ☐ Ecological, evolutionary & environmental sciences

For a reference copy of the document with all sections, see nature.com/documents/nr-reporting-summary-flat.pdf

Life sciences study design

All studies must disclose on these points even when the disclosure is negative.

Sample size	Sample size was determined based on previously published experiments where statistic differences were observed. (Mouse experiments; Nature Aging 2021;1:1117–1126. Nat Med 2018;24(8):1246-1256. Nat Med 2009;15(9):1082-1087. Cell culture experiments; Sci Rep 2022;12(1):6522. Nat Med 2018;24(8):1246-1256)
Data exclusions	Outliers and abnormal values probably due to technical error were excluded by Smirnov-Grubbs test with common threshold (α=0.05) in case that results follow a Gaussian distribution.
Replication	All data were from different biological replicates as indicated in each figure legend, and all findings were reproducible in different cohorts.
Randomization	All animals were randomly assigned to experimental and control groups by the investigator at baseline according to a pre-specified number in each replicate, after which key covariates (such as body weight and blood glucose levels) were confirmed to be equivalent between groups. In cell culture experiments, all wells of cultured cells were prepared at the same cell density and sequentially assigned to the different treatment groups.
Blinding	Mouse experiments for physiological, metabolic, and pathological analysis were blinded. Other experiments including in vitro and ex vivo studies, investigators were blinded to allocation during experiments and outcome assessments.

Reporting for specific materials, systems and methods

We require information from authors about some types of materials, experimental systems and methods used in many studies. Here, indicate whether each material, system or method listed is relevant to your study. If you are not sure if a list item applies to your research, read the appropriate section before selecting a response.

Materials & experimental systems

n/a	Involved in the study
☐	☒ Antibodies
☐	☒ Eukaryotic cell lines
☒	☐ Palaeontology and archaeology
☐	☒ Animals and other organisms
☒	☐ Clinical data
☒	☐ Dual use research of concern

Methods

n/a	Involved in the study
☒	☐ ChIP-seq
☐	☒ Flow cytometry
☒	☐ MRI-based neuroimaging

Antibodies

Antibodies used

[1st Antibodies]
Fcγ blocker(Rat Anti-Mouse CD16/CD32 antibody) antibody: used in FACS (1:100), Clone 2.4G2, C/N 553141, BD, Lot 0148675
BV421-conjugated anti-mouse CD31 antibody: used in FACS (1:100), Clone 390, C/N 102424, BioLegend, Lot B347937
PE/Cy7-conjugated anti-mouse CD45 antibody: used in FACS (1:100), Clone 30-F11, C/N 103114, BioLegend, Lot B308464
BB515-conjugated anti-mouse Cd11b antibody: used in FACS (1:100), Clone M1/70, C/N 564454, BD, Lot 0184624
BB700-conjugated anti-mouse Cd11b antibody: used in FACS (1:100), Clone M1/70, C/N 566416, BD, Lot 1350969
PE-conjugated anti-mouse CD3ε antibody: used in FACS (1:100), Clone 145-2C11, C/N 100302, BioLegend, Lot B346465
APC/Cy7-conjugated anti-mouse Nk1.1 antibody: used in FACS (1:100),Clone PK136, C/N 108724, BioLegend, Lot B344744
BV650-conjugated anti-mouse CD19 antibody: used in FACS (1:100), clone 6D5, C/N 115541, BioLegend, Lot B347917
BB700-conjugated anti-mouse Cd4 antibody: used in FACS (1:100), Clone RM4.5, C/N 566407, BD, Lot 1288744
Pacific Blue-conjugated anti-mouse CD8a antibody: used in FACS (1:100), Clone 53-6.7, C/N 100725, BioLegend, Lot B340544
BV711-conjugated anti-mouse CD274(Pd-l1) antibody: used in FACS (1:100), Clone 10F.9G2, C/N 124319, BioLegend, Lot B344019
PE-conjugated anti-human CD274(PD-L1) antibody: used in FACS (1:100), Clone 29E.2A3, C/N 329706, BioLegend, Lot B366030
BV711-conjugated anti-mouse CD69 antibody: used in FACS (1:100), clone H1.2F3, C/N 104537, BioLegend, Lot B350675
anti-p53 antibody: used in western blotting (1:1000), Clone 1C12, C/N 2524, Cell Signaling, Lot 13
anti-p53 antibody: used in western blotting (1:1000) and immuno-staining (1:25), Clone CM5, C/N NCL-L-p53-CM5p, Leica, Lot 609638
anti-phpspho-AMPKα antibody: used in western blotting (1:1000), Clone 40H9, C/N 2535, Cell Signaling, Lot 8
anti-AMPKα antibody: used in western blotting (1:1000), Clone D5A2, C/N 5831, Cell Signaling, Lot 21
anti-αTubulin antibody: used in western blotting (1:5000), Clone 11H10, C/N 2125, Cell Signaling, Lot 11
Ultra-LEAF™ Armenian Hamster anti-CD3ε antibody: used for T-cell depletion (40μg per body), C/N 100360, BioLegend, Lot B315767
Ultra-LEAF™ Armenian Hamster isotype control IgG: used for negative control (40μg per body), C/N 400960, BioLegend, Lot B291372

[2nd antibodies]
Peroxidase AffiniPure Goat Anti-Rabbit IgG (H+L): used in western blotting (1:5000), C/N 111-035-003,Jackson Immunoresearch Lot 150783
Peroxidase AffiniPure Goat Anti-mouse IgG (Light chain specific): used in western blotting (1:5000), C/N 111-035-174, Jackson Immunoresearch, Lot 152548
Biotin-SP (long spacer) AffiniPure™ Donkey Anti-Rabbit IgG (H+L): used in immuno-staining(1:25), C/N 711-065-152, Jackson Immunoresearch, Lot 133152

Validation

Fcγ blocker(553141, BD): Detected mouse CD16/31(Fcγ receptor) in FACS stated on the manufacturer's website.
BV421-conjugated anti-mouse CD31 antibody (102424, BioLegend): detected mouse CD31 in FACS stated on the manufacturer's website.
PE/Cy7-conjugated anti-mouse CD45 antibody (103114, BioLegend): detected mouse CD45 in FACS stated on the manufacturer's website.
BB515-conjugated anti-mouse Cd11b antibody (564454, BD): detected mouse CD11b in FACS stated on the manufacturer's website.
BB700-conjugated anti-mouse Cd11b antibody (566416, BD): detected mouse CD11b in FACS stated on the manufacturer's website.
PE-conjugated anti-mouse CD3ε antibody (100302, BioLegend): detected mouse CD3ε in FACS stated on the manufacturer's website.
APC/Cy7-conjugated anti-mouse Nk1.1 antibody (108724, BioLegend): detected mouse Nk1.1 in FACS stated on the manufacture'rs website.
BV650-conjugated anti-mouse CD19 antibody (115541, BioLegend): detected mouse CD19 in FACS stated on the manufacturer's website.
BB700-conjugated anti-mouse CD4 antibody(566407, BD): detected mouse CD4 in FACS stated on the manufacturer's website.
Pacific Blue-conjugated anti-mouse CD8a antibody(100725, BioLegend): detected mouse CD8a in FACS stated on the manufacturer's website.
BV711-conjugated anti-mouse CD274(Pd-l1) antibody(124319, BioLegend): detected mouse Pd-l1 in FACS stated on the manufacturer's website.
PE-conjugated anti-human CD274(PD-L1) antibody (329706, BioLegend): detected human PD-L1 in FACS stated on the manufacturer's website.
BV711-conjugated anti-mouse CD69 antibody (, Biolegend): detected mouse CD69 in FACS stated on the manufacturer's website.
anti-p53 antibody (2524, Cell Signaling): detected mouse p53 in western blotting stated on the manufacture's website.
anti-p53 antibody (NCL-L-p53-CM5p, Leica): validated to detect mouse p53 in mouse fibroblast celline(3T3L1) with p53 overexpression.
anti-phpspho-AMPKα antibody (2535, Cell Signaling): detected mouse phpspho-AMPKα in western blotting stated on the manufacturer's website.
anti-AMPKα antibody (5831, Cell Signaling): detected mouse phpspho-AMPKα in western blotting stated on the manufacturer's website.
anti-αTubulin antibody (2125, Cell Signaling): detected mouse phpspho-AMPKα in western blotting stated on the manufacturer's

website.
Ultra-LEAF™ Armenian Hamster anti-CD3ε antibody (100360, BioLegend): measured cell population in mouse blood and white adipose tissue and confirmed decrease of T-cell fraction in FACS.
Ultra-LEAF™ Armenian Hamster isotype control IgG (400960, BioLegend): measured cell population in mouse blood and white adipose tissue in FACS.

Eukaryotic cell lines

Policy information about cell lines and Sex and Gender in Research

Cell line source(s)	Mouse ear-fibroblast: obtained from ear skin tissues of wild-type or CAG-tdTomato mice (C57BL/6 strain) at 4-week-old.<br>Human umbilical vein endothelial cell (HUVEC): purchased from Lonza (C/N cc-2517, Lot 0000439577).<br>IMR90 (Human fetal lung fibroblast): purchased from KAC (C/N EC85020204-F0, Lot 14F012).
Authentication	Mouse ear-fibroblast: verified by qPCR to detect mouse Rplp0 mRNA.<br>HUVEC: verified by qPCR and immunostaining of human CD31, and tube formation assays done by Lonza.<br>IMR90: verified by qPCR to detect human RPLP0 mRNA.
Mycoplasma contamination	Mouse ear-fibroblast: not tested.<br>HUVEC: certified to be micoplasma-negative by Lonza.<br>IMR90: certified to be micoplasma-negative by the distributer; European Collection of Cell Cultures.
Commonly misidentified lines (See ICLAC register)	None.

Animals and other research organisms

Policy information about studies involving animals; ARRIVE guidelines recommended for reporting animal research, and Sex and Gender in Research

Laboratory animals	C57BL/6 mice were purchased from SLC Japan (Shizuoka, Japan).<br>ApoE KO mice (C57BL/6 background) were obtained from the Jackson Laboratory.<br>Zmpste24-deficient mice were obtained from the Jackson Laboratory (MGI: 2158363).<br>p19Arf-DTR-luciferase mice (C57BL/6 background) were obtained from Sugimoto M.<br>CAG-tdTomato mouse (C57BL/6 background)were obtained from Abe M and their colleague.<br><br>Mice were maintained in pathogen-free facility at 20-26°C, 40-60% humidity, under 12-hour light and 12-hour dark regimen. Mice were imposed on a high fat diet (HFD32, CLEA Japan) or normal chow (CE-2, CLEA Japan or CRF-1, Oriental East Japan) from 4 to 16 weeks old, unless otherwise described in the figure legends. ApoE KO mice (C57BL/6 background) were obtained from the Jackson Laboratory and imposed to western diet (F2HFD1, Oriental Yeast, Japan) from 4 to 18 weeks old.
Wild animals	no wild animals were used in the study.
Reporting on sex	All mouse experiments except lifspan measurement in Zmpste 24-deficient mice were analyzed only in male mice.
Field-collected samples	No field collected samples were used in the study.
Ethics oversight	All of the animal experiments were conducted in compliance with the protocol reviewed by the Institutional Animal Care and Use Committee of Niigata University or Juntendo University and approved by their Presidents.

Note that full information on the approval of the study protocol must also be provided in the manuscript.

Flow Cytometry

Plots

Confirm that:

☒ The axis labels state the marker and fluorochrome used (e.g. CD4-FITC).

☒ The axis scales are clearly visible. Include numbers along axes only for bottom left plot of group (a 'group' is an analysis of identical markers).

☒ All plots are contour plots with outliers or pseudocolor plots.

☒ A numerical value for number of cells or percentage (with statistics) is provided.

Methodology

Sample preparation	White adipose tissue, Aorta, matrigel, fibroblast from ears were excised, minced and digested with digesting solution (2mg/ml collagenase type II (Worthington), and 1mM calcium chloride (CaCl2) in phosphate-buffered saline (PBS)) for 20~30 minutes at 37 °C. Mouse spleen and bone marrow were excised, and their cell aggregation were mashed gently in PBS. Cell suspension was subsequently filtered through a nylon mesh (40µm), and red blood cell lysis was achieved with an ammonium chloride-based lysing buffer (Pharm Lyse, 555899, BD). Cultured HUVECs were detached and collected from cell-culture

	dishes by incubation in 0.05% Trypsin - ethylenediammine tetraacetic acid (EDTA) solution at 37°C for 3 minutes. Cells were resuspended in PBS supplemented with 1% fetal bovine serum (FBS) and 5mM EDTA for FACS analysis, or in DMEM containing 10% FBS and 1% PS for cell culture.
Instrument	Data collection was performed by a spectral cell analyzer ID7000 (Sony) or a cell sorter SH800S (Sony).
Software	Data were collected and analyzed with ID7000 software (version 1.1.0.11041, Sony) or SH800S cell sorter software (version 2.1.6, Sony), and FlowJo (version 10.8.1, BD)
Cell population abundance	Immune cell fractions: calculated as percentage per total cell number in SVF from gWAT, spleen and bone marrow.<br>SPiDER-β-gal+ and/or Pd-l1+ cell fractions: calculated as percentage per total cell number in SVF from gWAT and aorta.<br>tdTomato+ ear-fibroblasts in Matrigel: calculated as cell counts per Matrigel weight (per mg).<br>PD-L1 / Pd-l1 abundance in HUVECs : calculated geometrical mean fluorescent intensity of PD-L1 or Pd-l1 antibodies.
Gating strategy	Isolated cells from the SVF fraction in gWAT, aorta, spleen, or bone marrow were subjected to FACS analysis for SPiDER-β-gal or cell surface makers. The number of SPiDER+ senescent cells, PD-L1+SPiDER+ cells, macrophages (CD45+ CD11b+), T cells (CD45+ CD11b– CD3ε+), CD4+ T cells (CD45+ CD11b– CD3ε+ CD4+), CD8+ T cells (CD45+ CD11b– CD3ε+ CD8a+), activated CD8+ T cells (CD45+ CD11b– CD3ε+ CD8a+ CD69+), B cells (CD45+ CD11b– CD19+) and NK cells (CD45+ CD11b– NK1.1+) was examined. For bone marrow derived cells, the number of myeloid cells (CD45+ CD11b+), lymphoid cells (CD45+ CD11b–) was examined. In HUVECs, mean fluorescent intensity from PD-L1 antibody was measured.

☒ Tick this box to confirm that a figure exemplifying the gating strategy is provided in the Supplementary Information.
