# Supplementary figures and images for: SGLT2 inhibition eliminates senescent cells and alleviates pathological aging
Source: Nat Aging. 2024 May 30;4(7):926–38. doi: 10.1038/s43587-024-00642-y (PMC11257941; doi:10.1038/s43587-024-00642-y)

Figure 1e

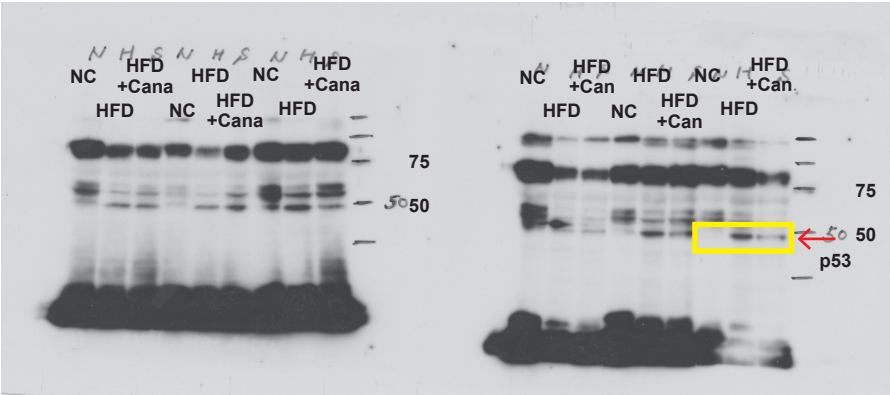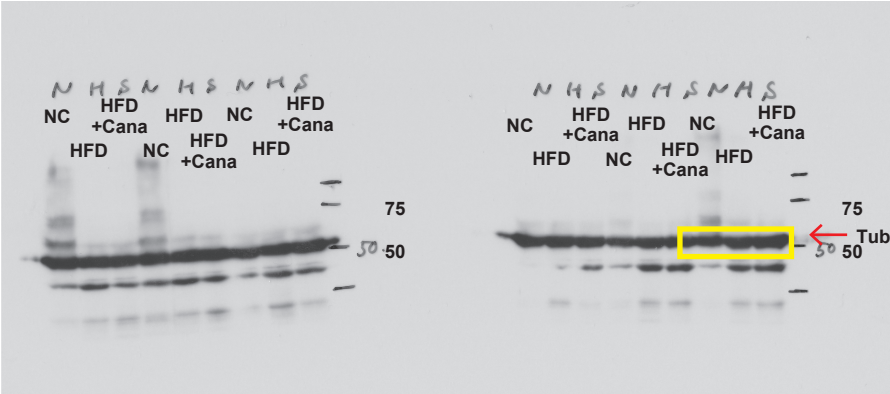

Supplement: Supplementary file 5 — Unprocessed western blots. [file 43587_2024_642_MOESM5_ESM.pdf]

Figure 2b

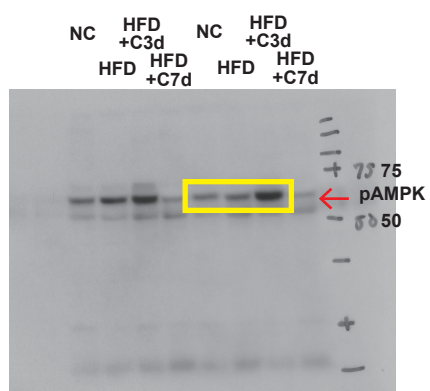

Used for main figure

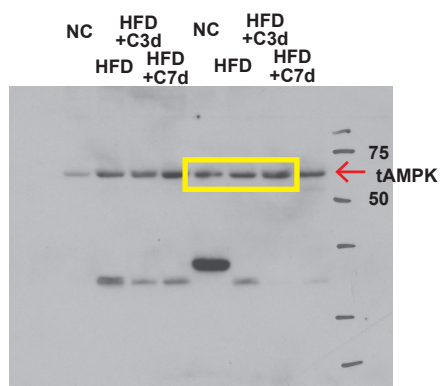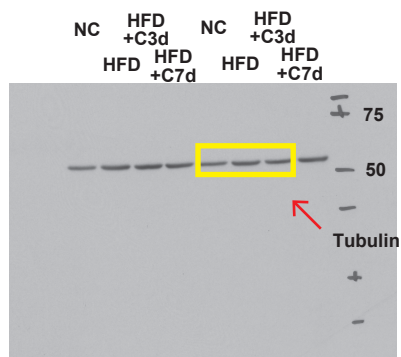

Supplement: Supplementary file 7 — Unprocessed western blots. [file 43587_2024_642_MOESM7_ESM.pdf]

Extended Data Figure 2f

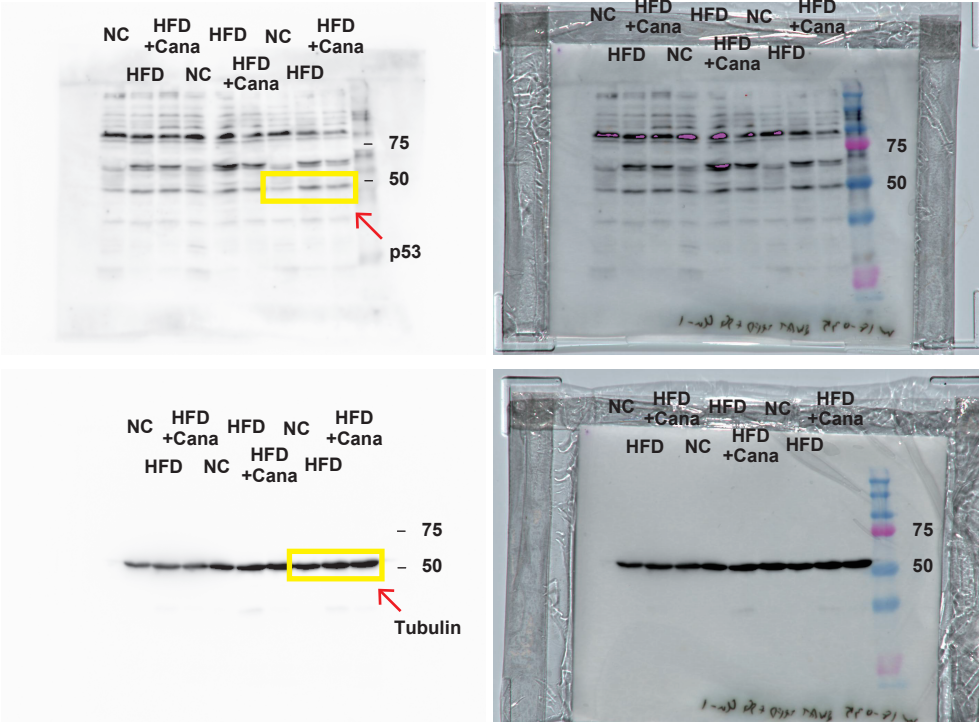

Supplement: Supplementary file 12 — Unprocessed western blots. [file 43587_2024_642_MOESM12_ESM.pdf]

Extended Data Figure 3e

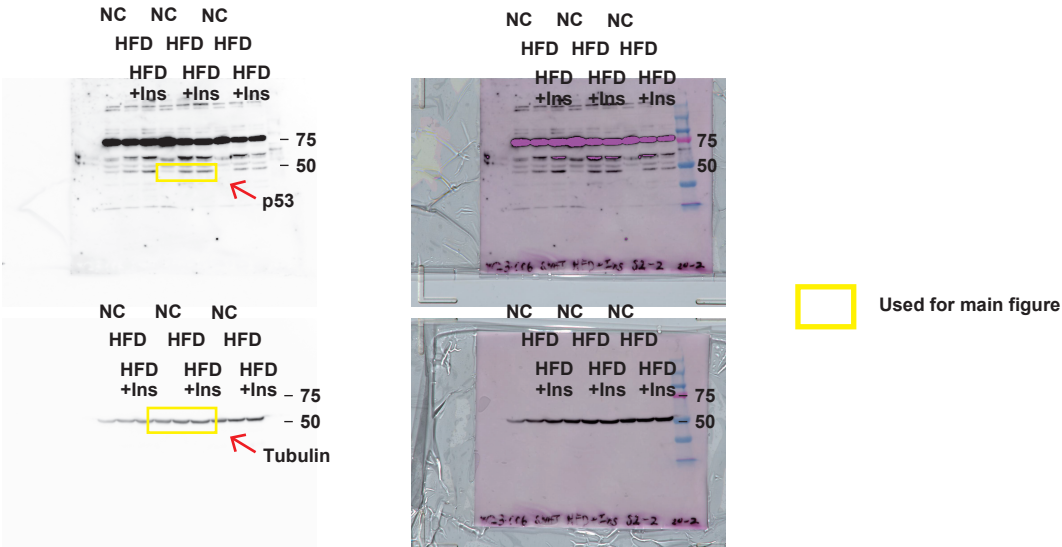

Supplement: Supplementary file 14 — Unprocessed western blots. [file 43587_2024_642_MOESM14_ESM.pdf]

Extended Data Figure 5b

Liver

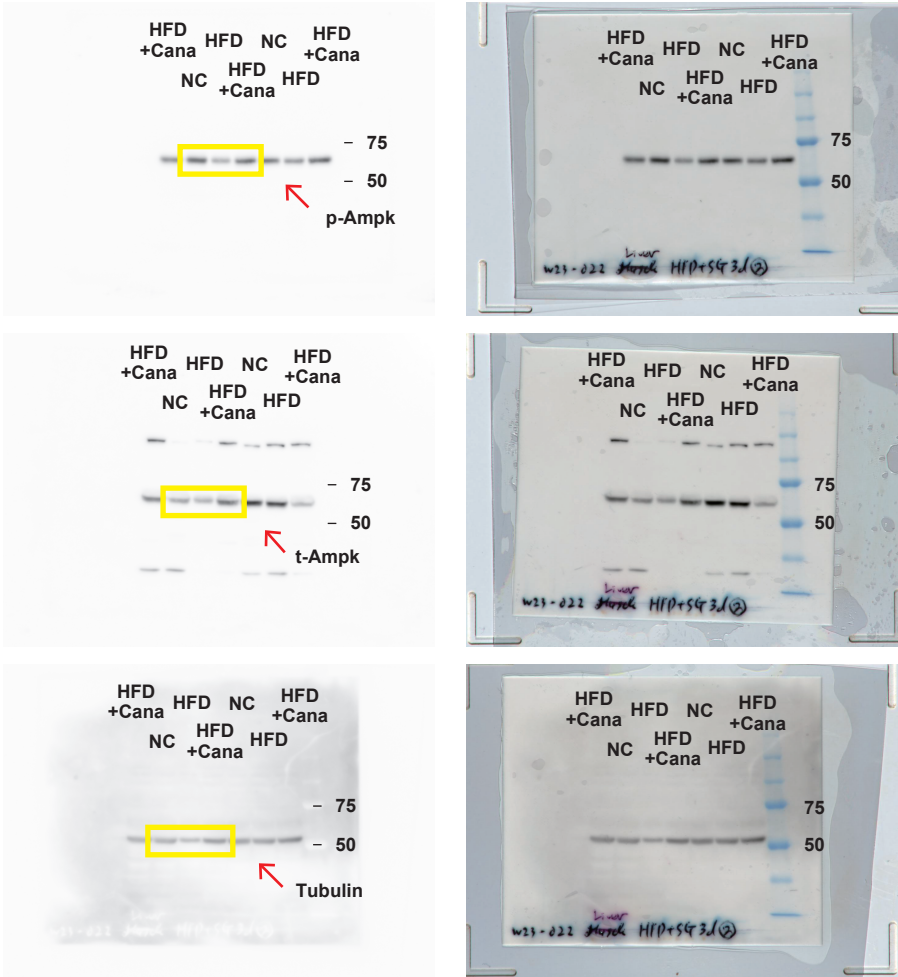

Used for main figure

Muscle

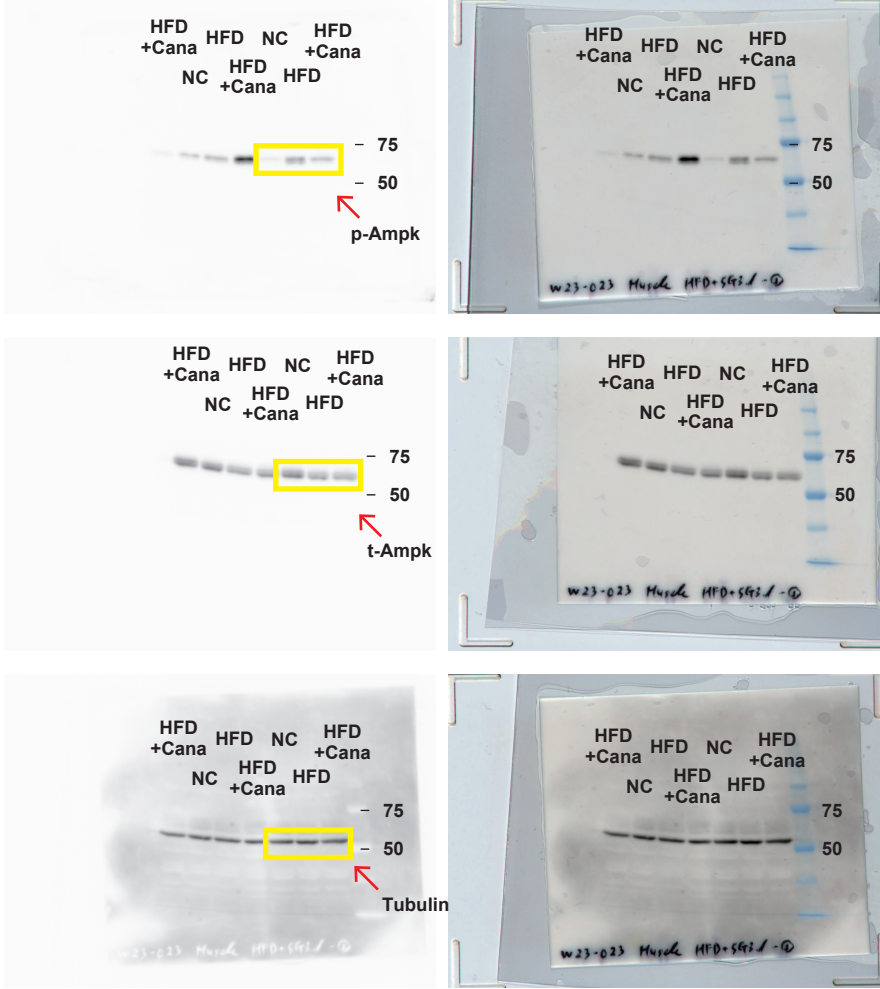

Used for main figure

Supplement: Supplementary file 17 — Unprocessed western blots. [file 43587_2024_642_MOESM17_ESM.pdf]
